# A universal 6iL/E4 culture system for deriving and maintaining embryonic stem cells across mammalian species

**DOI:** 10.1038/s41422-026-01276-y

**Published:** 2026-07-13

**Authors:** Duo Wang, Hao Ming, Dongshan Yang, Xiang Cui, Zachary Freeman, Li-Kuang Tsai, Zhuying Wei, Liu Liu, Giovanna Nascimento Scatolin, Brian Bennett, Xiukun Wang, Kimberly Yau, Litao Tao, Xinyi Tong, Shuling Wang, Kai-Xuan Shi, Denis Evseenko, Ben Van Handel, Longhua Guo, Xiaoting Dai, Youcai Xiong, Bingjing Zhang, Yinjuan Wang, Rajan Iyyappan, Oscar Alejandro Ojeda-Rojas, Guang Hu, Lynda McGinnis, Richard Paulson, Daniel Mckim, Xiangbo Kong, Xiaofeng Xia, Jifeng Zhang, Y. Eugene Chen, Zongliang Jiang, Jie Xu, Qi-Long Ying

**Affiliations:** 1 Eli and Edythe Broad Center for Regenerative Medicine and Stem Cell Research at USC, Department of Stem Cell Biology and Regenerative Medicine, Keck School of Medicine, University of Southern California, Los Angeles, CA USA; 2 Center for Advanced Models Translational Sciences and Therapeutics, University of Michigan Medical School, Ann Arbor, MI USA; 3 Department of Animal Sciences, Genetics Institute, Institute of Food and Agricultural Sciences, University of Florida, Gainesville, FL USA; 4 Transgenic Animal Model Core, University of Michigan Medical School, Ann Arbor, MI USA; 5 Gilbert S. Omenn Department of Computational Medicine and Bioinformatics, Ann Arbor, MI USA; 6 Epigenetics and RNA Biology Laboratory, National Institute of Environmental Health Sciences, Durham, NC USA; 7 Department of Obstetrics and Gynecology, Keck School of Medicine, University of Southern California, Los Angeles, CA USA; 8 Department of Biomedical Sciences, School of Medicine, Creighton University, Omaha, NE USA; 9 Transgenic Mouse Facility, University of California, Irvine, CA USA; 10 Department of Orthopaedic Surgery, Keck School of Medicine of the University of Southern California, Los Angeles, CA USA; 11 Department of Molecular & Integrative Physiology, University of Michigan, Ann Arbor, MI USA; 12 School of Animal Sciences, AgCenter, Louisiana State University, Baton Rouge, LA USA; 13 Department of Large Animal Clinical Sciences, University of Florida, Gainesville, FL USA; 14 ATGC Inc., Philadelphia, PA USA

**Keywords:** Stem cells, Pluripotency, Self-renewal

## Abstract

The derivation of authentic embryonic stem cells (ESCs) across mammalian species remains a major challenge. Here, we report the development of a defined, serum-free culture system, termed 6iL/E4, that enables the derivation and long-term self-renewal of ESCs across diverse mammalian species. Through systematic dissection of signaling pathways, we identified conserved regulatory modules involving GSK3α, WNT, STAT3, PDGFR, and MEK/ERK signaling. The optimized 6iL/E4 conditions support stable derivation and expansion of ESCs from mouse, rat, rabbit, and bovine embryos. For rabbit, ESC derivation required supplementation with the LATS inhibitor TDI-011536 (TDI), and 6iL/TDI-cultured rabbit ESCs exhibited chimera-forming capability. In bovine ESCs, inducible expression of *Klf2* and *Nanog* reinforced pluripotency and promoted in vivo chimeric contribution. Importantly, we demonstrated that 6iL robustly establishes and maintains human pluripotent stem cells in a naïve-like state. These findings reveal conserved principles underlying ESC self-renewal across divergent mammalian species and provide a universal platform for cross-species stem cell research, disease modeling, and biotechnological applications.

## Introduction

In mammals, embryonic stem cells (ESCs) are pluripotent stem cells (PSCs) derived from the inner cell mass (ICM) of preimplantation embryos. They possess the capacity for unlimited self-renewal and can differentiate into all somatic and germ cell lineages in vitro and in chimeric animals.^1,2^ Authentic mammalian ESCs capable of contributing to chimeras and transmitting through the germline have been successfully established only in mice^1,2^ and rats.^3,4^ Despite decades of effort, comparable germline-competent ESCs have not been derived from other mammalian species.

The successful derivation of mouse ESCs (mESCs) initially relied on culture systems using mitotically inactivated mouse embryonic fibroblast (MEF) feeders together with leukemia inhibitory factor (LIF) and fetal bovine serum (FBS) or bone morphogenetic protein (BMP).^5,6^ A major breakthrough came with the development of the 2i condition, consisting of the MEK1/2 inhibitor PD0325901 (PD03) and the pan-GSK3 inhibitor CHIR99021 (CHIR), which stabilizes naïve pluripotency by suppressing differentiation-inducing pathways.^7^ The 2i or 2i/LIF system markedly improved derivation efficiency across mouse strains and enabled, for the first time, the establishment of germline-competent rat ESCs (rESCs).^3,4^ However, these conditions have not robustly supported the derivation of bona fide ESCs from non-rodent mammals. Whether a universal culture system can sustain authentic ESCs across diverse mammalian species remains an unresolved and fundamental question.

Among non-rodent mammals, the rabbit represents a valuable biomedical model owing to its short gestation period, relatively large litter size, and physiological similarities to humans.^8,9^ Phylogenetically closer to humans than mice, rabbits are widely used in pulmonary, cardiovascular, metabolic, and immunological research.^9^ Similarly, cattle provide an informative large-animal model for human preimplantation development because bovine and human embryos share striking similarities during early embryogenesis.^10–12^ In addition, rabbit ESCs (rabESCs) and bovine ESCs (bESCs) have substantial agricultural and biotechnological applications. However, authentic ESCs from rabbits and cattle, comparable to rodent naïve ESCs, have not yet been established. Previously reported lines generally fail to meet stringent functional criteria, particularly chimera and germline contribution, and are often described as “ES-like” cells.^13–15^ Although germline-competent rabbit induced pluripotent stem cells (iPSCs) have recently been generated via transcription factor-mediated reprogramming,^16^ these cells require sustained ectopic transgene expression and specialized culture conditions. A stable, transgene-free, and broadly applicable ESC derivation system for rabbit and bovine species remains lacking. Similarly, bovine “primed” ESCs^17^ and expanded pluripotent stem cells (EPSCs)^18^ have not demonstrated germline-competent chimera formation in vivo. These limitations likely reflect suboptimal culture environments and an incomplete understanding of the signaling networks that govern pluripotency in non-rodent mammals.

We hypothesized that the core regulatory logic underlying ESC self-renewal is evolutionarily conserved across mammals, even if specific pathway outputs appear species-dependent. The successful application of 2i-based conditions to both mouse and rat ESCs^3,4,7^ supports this notion. Nevertheless, reported differences in signaling responses have raised questions regarding conservation. For example, nuclear β-catenin promotes self-renewal in rodent ESCs but has been reported to induce differentiation in human ESCs (hESCs).^4,7,19^ We propose that such differences reflect context-dependent pathway modulation or feedback architecture rather than fundamental divergence of pluripotency networks. Consistent with this view, we recently demonstrated that selective inhibition of GSK3α using BRD0705 sustains pluripotent and adult stem cell self-renewal through a β-catenin-independent mechanism, revealing previously unappreciated conserved signaling modules.^20^ These findings suggest that refinement of pathway targeting, rather than wholesale pathway substitution, may enable cross-species stabilization of naïve pluripotency.

In the present study, we sought to establish a universal ESC culture system by systematically targeting conserved signaling pathways. We interrogated the roles of STAT3, WNT, GSK3α, PDGFR, and MEK/ERK signaling in rabbit and bovine embryo-derived cells and optimized a defined basal medium, converting N2B27 into a simplified four-component formulation termed E4. These efforts culminated in the development of a fully defined, serum-free 6iL/E4 culture system. Using this platform, we successfully derived rabbit and bovine ESCs. The 6iL/E4 condition also enables the derivation of mESCs, maintains rESCs, and supports the long-term self-renewal of naïve-like human iPSCs (hiPSCs) and hESCs. Importantly, 6iL-derived rabESCs exhibited chimera-forming capacity, and 6iL-derived bESCs transcriptionally corresponded to an early blastocyst-stage pluripotent state along the developmental trajectory.

## Results

### Development of a defined 6iL/E4 culture system for rabbit and bovine ESC derivation

The 2i/N2B27 system efficiently supports the derivation and maintenance of mouse and rat ESCs.^3,4,7^ We initially applied this condition to bovine and rabbit embryos; however, all embryo-derived cells underwent overt differentiation or cell death (Supplementary information, Fig. S1a–d). These results suggest that components of the 2i/N2B27 system may actively promote differentiation in non-rodent species.

Given the complex composition of N2B27 and its known propensity to induce neural differentiation in mESCs in the absence of 2i,^21^ we hypothesized that certain additives might compromise pluripotency maintenance. To test this, we systematically evaluated which individual N2B27 components were required for mESC self-renewal under 2i conditions. N2B27 comprises a 1:1 mixture of DMEM/F12 and Neurobasal media supplemented with N2 and B27, both of which contain multiple bioactive components. We therefore reconstructed the medium beginning with DMEM/F12 and Neurobasal supplemented only with insulin, transferrin, and bovine serum albumin (BSA), the core shared components of N2 and B27, and sequentially reintroduced the omitted additives. This screening identified sodium selenite as the sole additional component required to sustain long-term mESC self-renewal (Supplementary information, Fig. S1e, f). We designated this minimal 4-component formulation “E4” medium. Importantly, 2i/E4 robustly supported long-term self-renewal of both mESCs and rESCs (Supplementary information, Fig. S1g, h). rESCs cultured in 2i/E4 displayed improved colony morphology and reduced cell death compared with those cultured in 2i/N2B27 (Supplementary information, Fig. S1h, i). Notably, unlike N2B27, E4 did not induce neural differentiation of mESCs in the absence of 2i (Supplementary information, Fig. S1l–o). Furthermore, mESC-derived neural progenitors failed to survive in E4 (Supplementary information, Fig. S1j–o), indicating that this simplified medium disfavors neural lineage progression. Long-term culture of mESCs and rESCs in E4 under conventional naïve conditions, including 2i or 2i/LIF, confirmed preservation of morphology and molecular identity comparable to cells maintained in N2B27 (Supplementary information, Fig. S2). Collectively, these results establish E4 as a simplified, defined basal medium that eliminates differentiation-promoting additives while fully supporting rodent naïve ESCs.

**Fig. 1:**
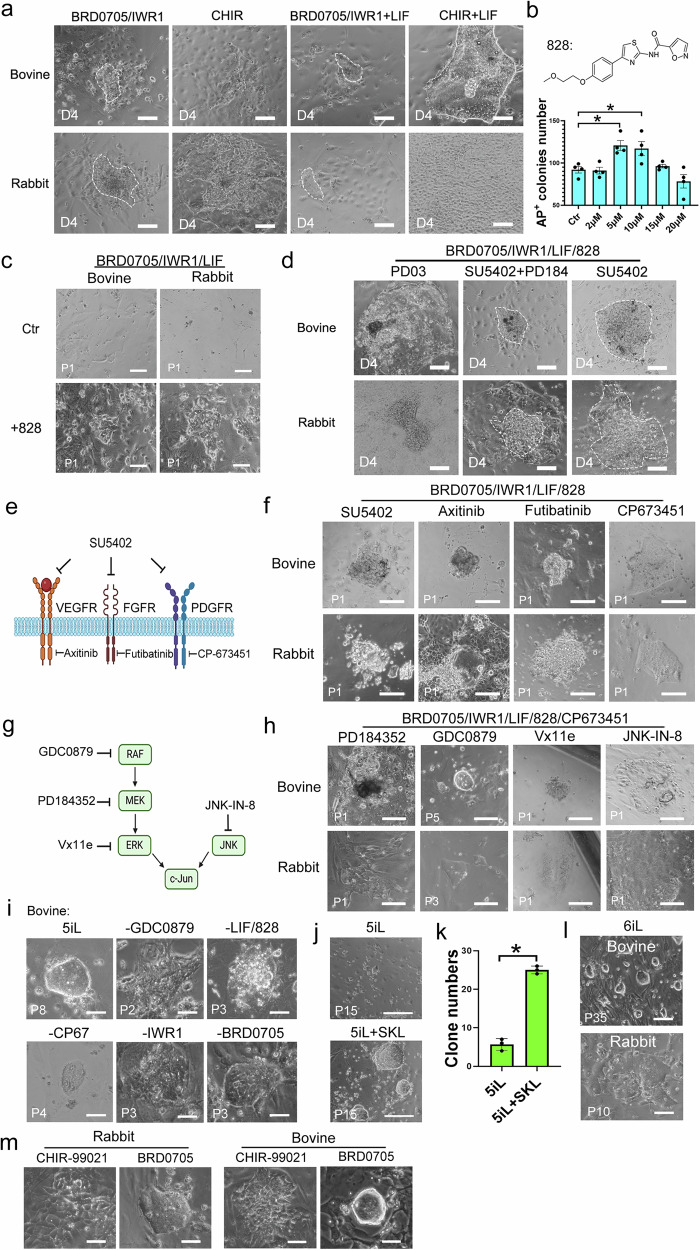
Optimization and evaluation of culture conditions for deriving and expanding bovine and rabbit ESCs. **a** Representative phase-contrast images of bovine and rabbit ICMs cultured under different conditions on day 4. Scale bars, 100 μm. Dashed outlines indicate undifferentiated ICM outgrowths. **b** Chemical structure of compound 828 and quantification of alkaline phosphatase-positive (AP^+^) colonies in mESC cultures treated with 20 ng/mL LIF and varying concentrations of 828. Data are presented as mean ± SEM, with statistical significance indicated (*P < 0.05). **c** Representative morphology of passage 1 (P1) cells derived from bovine and rabbit ICMs cultured in BRD0705/IWR1/LIF with or without 828. Scale bars, 100 μm. **d** Representative outgrowth morphology of bovine and rabbit ICMs cultured for 4 days in BRD0705/IWR1/LIF/828 conditions with the indicated signaling pathway inhibitors. Scale bars, 100 μm. Dashed outlines indicate undifferentiated ICM outgrowths. **e** Schematic illustration of the targets of SU5402 (created with BioRender.com). **f** Representative morphology of P1 ESCs derived from bovine and rabbit embryos and cultured in BRD0705/IWR1/LIF/828 supplemented with SU5402, axitinib, futibatinib, or CP67. Scale bars, 100 µm. **g** Diagram of the MEK/ERK signaling cascade and the corresponding small-molecule inhibitors used in this study: GDC0879 (RAF), PD184352 (MEK), Vx-11e (ERK), and JNK-IN-8 (JNK) (created with BioRender.com). **h** Phase-contrast images showing the effects of MEK/ERK pathway inhibitors on bESC and rabESC derivation. ESCs were cultured in E4 medium supplemented with BRD0705, IWR1, LIF, 828, and CP67, with individual MEK/ERK pathway inhibitors added separately. Scale bars, 100 µm. **i** Representative images of bESCs derived from bovine blastocysts and cultured in 5iL/E4 medium, with or without withdrawal of the indicated components. Scale bars, 50 µm. **j** Phase-contrast images of bESCs cultured under 6iL conditions, consisting of 5iL medium supplemented with the additional small molecule SKL2001 (SKL). Scale bars, 200 µm. **k** Quantification of bESC colony numbers in 5iL medium with or without SKL2001. Data are presented as mean ± SEM. *P < 0.05. **l** Phase-contrast images of P35 bESCs derived from blastocysts and P10 rabESCs derived from morula-stage embryos in 6iL/E4 medium. Scale bars, 100 µm. **m** Phase-contrast images of bESCs and rabESCs derived in IWR1/CP67/GDC0879/LIF/828/SKL2001 supplemented with either CHIR or BRD0705. Scale bars, 50 µm.

With E4 as a defined basal platform, we next screened small molecules and growth factors to identify culture conditions capable of supporting ESC derivation from bovine and rabbit embryos. Because rodent naïve pluripotency depends on canonical WNT/β-catenin signaling activation and MEK/ERK inhibition,^3,4,7^ we first assessed the role of WNT signaling. In rodent ESCs, GSK3 inhibition stabilized β-catenin and promoted its nuclear translocation to support self-renewal.^7^ In contrast, β-catenin activation in naïve hESCs has been reported to induce differentiation and apoptosis.^19^ Consistent with the human phenotype, treatment with the pan-GSK3 inhibitor CHIR induced rapid differentiation of bovine and rabbit ICM outgrowths (Fig. 1a; Supplementary information, Fig. S1a and Table S1).

We therefore tested BRD0705, a selective GSK3α-specific inhibitor,^22^ in combination with the tankyrase inhibitor IWR1.^23^ This combination more effectively suppressed the expansion of non-ESC-like cells than did CHIR (Fig. 1a; Supplementary information, Fig. S3a and Table S1), consistent with our recent study demonstrating that BRD0705/IWR1 supports self-renewal across multiple pluripotent states.^20^ However, BRD0705/IWR1 alone was insufficient to support sustained propagation of rabbit or bovine ESCs, indicating that additional pathways are required.

We next evaluated activation of the STAT3 pathway. Addition of LIF to BRD0705/IWR1 enhanced the formation of ESC-like colonies from bovine and rabbit ICM outgrowths (Fig. 1a; Supplementary information, Table S1). To further potentiate STAT3 signaling, we screened additional activators and identified 828,^24^ a small molecule that synergized with LIF to enhance mESC colony formation (Fig. 1b; Supplementary information, Fig. S3c, d). Although the combination of BRD0705/IWR1/LIF/828 enabled the transient emergence of ESC-like colonies from rabbit and bovine embryos, these colonies eventually developed vacuole-like structures and underwent differentiation. Nevertheless, inclusion of 828 improved short-term colony stability and permitted survival through at least one passage (Fig. 1c).

Given the central role of FGF/MEK/ERK signaling in pluripotency regulation,^7^ we next screened pathway inhibitors in the context of BRD0705/IWR1/LIF/828. SU5402, a multi-kinase inhibitor targeting VEGFR, FGFR, and PDGFR, effectively suppressed non-ICM outgrowths and enhanced ESC-like colony formation (Fig. 1d; Supplementary information, Table S2). To identify the relevant target, we tested selective inhibitors: axitinib for VEGFR, futibatinib for FGFR, and CP673451 (CP67) for PDGFR. Only CP67 supported stable ESC colony formation after passaging (Fig. 1e, f; Supplementary information, Table S2). Moreover, in rESCs, CP67 fully substituted for SU5402 when combined with CHIR and PD184352, implicating PDGFR inhibition as the principal mediator of SU5402 activity in ESC maintenance (Supplementary information, Fig. S3e, f).

Incorporation of CP67 yielded a five-component condition (BRD0705/IWR1/LIF/828/CP67) that permitted limited passaging (approximately 2 passages) of rabbit and bovine ESCs. To enhance stability, we screened additional MEK/ERK pathway inhibitors, including GDC0879, a BRAF inhibitor; PD184352, a MEK inhibitor; Vx-11e, an ERK inhibitor; and JNK-IN-8, a JNK inhibitor (Fig. 1g). GDC0879 uniquely enabled extended propagation of ESC-like colonies from both species (Fig. 1h; Supplementary information, Table S3). This optimized six-component combination, comprising BRD0705, IWR1, LIF, 828, CP67, and GDC0879, was designated “5iL”. Withdrawal of any single component abolished bESC self-renewal (Fig. 1i), demonstrating that these pathways function cooperatively.

Although 5iL enabled efficient derivation of rabESCs and bESCs, bESCs progressively lost proliferative capacity after ~15 passages. To overcome this limitation, we performed an additional small-molecule screen and identified SKL2001, previously characterized as a WNT/β-catenin pathway activator.^25^ SKL2001 markedly enhanced bESC colony formation and supported long-term maintenance of rabESCs (Fig. 1j, k). Notably, mechanistic analysis revealed that SKL2001 promotes mESC self-renewal independently of β-catenin (Supplementary information, Fig. S3g, h), indicating a noncanonical mode of action.

Incorporation of SKL2001 yielded the final “6iL” cocktail — BRD0705, IWR1, CP67, GDC0879, SKL2001, 828, and LIF — used in combination with E4 basal medium (6iL/E4) for ESC derivation (Fig. 1l). Importantly, substituting BRD0705 with CHIR in this optimized system resulted in widespread differentiation in both rabbit and bovine cultures (Fig. 1m), underscoring the distinct response of non-rodent ESCs to canonical WNT/β-catenin activation and highlighting the importance of selective GSK3α inhibition without canonical β-catenin activation.^20^

Together, these data establish 6iL/E4 as a robust, defined culture system that enables efficient derivation and sustained maintenance of rabESCs and bESCs, revealing conserved yet context-dependent signaling requirements underlying mammalian pluripotency.

### Stable and long-term maintenance of rodent ESCs in 6iL/E4

**Fig. 2:**
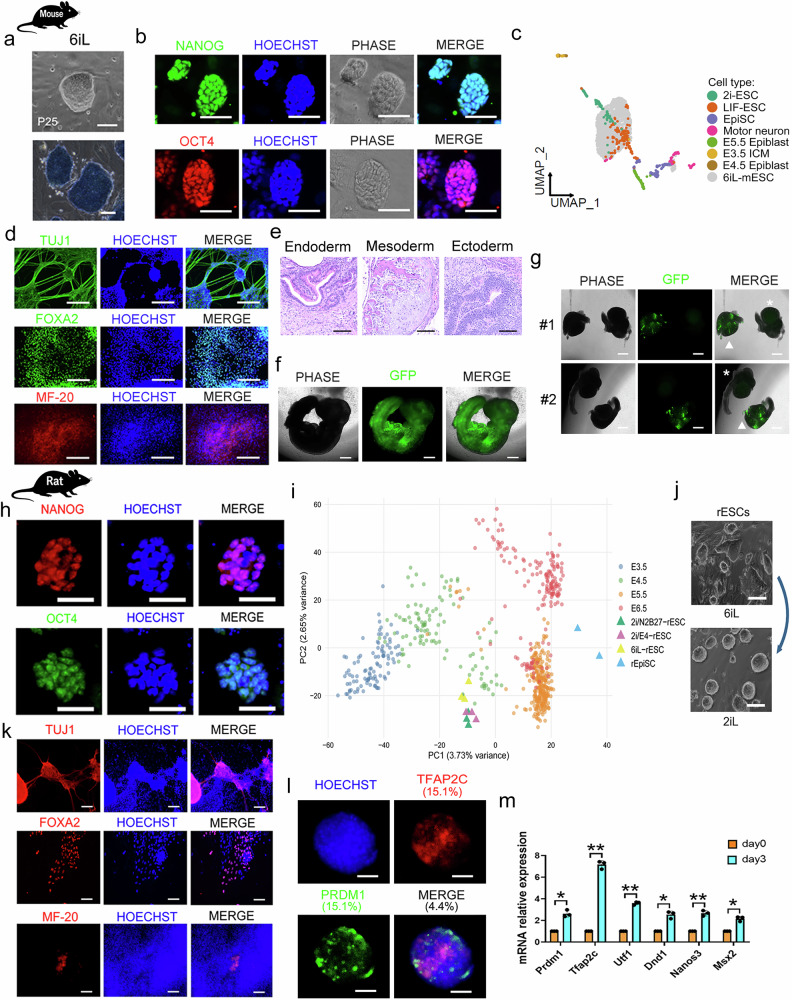
Characterization of mESCs and rESCs derived and maintained in 6iL. **a** Top: representative phase-contrast images showing ESCs cultured in 6iL for 25 passages. Scale bars, 100 µm. Bottom: AP staining of ESC colonies derived in 6iL. Scale bar, 50 µm. **b** Representative immunofluorescence (IF) images of 6iL-derived ESCs (P15). Green indicates NANOG; red indicates OCT4; blue indicates Hoechst. Scale bars, 100 µm. **c** UMAP projection showing transcriptional relationships among PSC states and embryonic reference populations. Each dot represents a single cell colored by cell type, including 2i-ESCs, LIF-ESCs, EpiSCs, motor neurons, E5.5 epiblast, E3.5 ICM, E4.5 epiblast, and 6iL-mESCs, illustrating the positioning of 6iL-mESCs relative to embryonic developmental stages and PSC states. Reference dataset for this figure: GSE74155.^59^ **d** Representative IF images of EB outgrowths derived from 6iL-mESCs stained for multilineage differentiation markers. Scale bars, 200 µm. **e** Representative hematoxylin and eosin (H&E) staining of teratomas derived from 6iL-mESCs (P30), showing differentiated tissues representative of the three germ layers: endoderm, mesoderm, and ectoderm. Scale bars, 100 µm. **f** Representative bright-field and fluorescence images of E9.5 chimera generated by injecting GFP-labeled 6iL-mESCs into blastocysts. Scale bars, 500 µm. **g** Representative images of gonads isolated from E13.5 embryos. Arrowheads indicate gonads derived from chimeric embryos generated using GFP-labeled 6iL-mESCs, whereas asterisks denote gonads from wild-type control embryos. GFP fluorescence shows the distribution of cells differentiated from GFP-labeled 6iL-mESCs within the gonads. Scale bars, 500 µm. **h** Representative IF images of rESCs cultured in 6iL, showing expression of pluripotency markers NANOG (red) and OCT4 (green). Hoechst (blue) marks nuclei. Scale bars, 50 µm. **i** PCA plot showing transcriptomic relationships between mouse embryonic stages (E3.5–E6.5) and stem cell populations, including 2i/N2B27-rESCs, 2i/E4-rESCs, 6iL-rESCs, and rEpiSCs. Reference datasets for mouse E3.5–E6.5 embryos were obtained from GSE100597,^60^ and rEpiSC reference data were obtained from GSE178701.^57^ **j** Representative images of rESCs cultured in 6iL for 6 passages and of 6iL-rESCs following transition to 2i conditions for two additional passages. Scale bars, 100 µm. **k** IF analysis of EB outgrowths derived from 6iL-rESCs, showing expression of markers representing the three germ layers: TUJ1 (ectoderm), FOXA2 (endoderm), and MF-20 (mesoderm). Nuclei are counterstained with Hoechst (blue). Scale bars, 100 µm. **l** IF staining of TFAP2C (red) and PRDM1 (green) in EBs formed from 6iL-rESCs. Scale bars, 50 µm. **m** qRT-PCR analysis of PGC marker gene expression in PGC-LCs derived from 6iL-rESCs. Data are presented as mean ± SEM. *P < 0.05, **P < 0.01.

To determine whether the 6iL/E4 system supports ESC maintenance across both rodent and non-rodent mammals, we evaluated its ability to derive and maintain rodent ESCs. mESC lines were successfully derived from blastocysts under 6iL/E4 (Fig. 2a). These 6iL-mESCs exhibited normal karyotypes (2n = 40, 77.5%, 31/40 metaphases at passage 35; Supplementary information, Fig. S4a), retained alkaline phosphatase (AP) activity (Fig. 2a), and uniformly expressed the core pluripotency factors NANOG and OCT4 (Fig. 2b), indicating stable maintenance of an undifferentiated state.

Single-cell RNA sequencing (scRNA-seq) analysis revealed that 6iL-mESC cultures consisted predominantly of an ESC population (94.66%), with only a minor primed-like fraction (5.35%) (Supplementary information, Fig. S4b–d). Uniform manifold approximation and projection (UMAP) analysis integrating embryonic reference datasets positioned the dominant 6iL-mESC population between embryonic day 4.5 (E4.5) and E5.5 and demonstrated substantial overlap with conventional mESCs cultured in 2i/LIF or LIF/serum (Fig. 2c). These findings indicate that core mESC features are preserved under 6iL conditions. Consistently, qRT-PCR analysis showed that expression of ESC-associated genes, including *Tfcp2l1*, *Rex1*, and *Sox2*, was comparable to that observed in 2i/LIF-cultured mESCs (Supplementary information, Fig. S4e). In contrast, 6iL-mESCs lacked expression of the primed marker *Foxa2* and exhibited reduced levels of the lineage-associated genes *T* and *Gata4* (Supplementary information, Fig. S4e), further supporting maintenance of the ESC transcriptional program.

Interestingly, bulk RNA sequencing (RNA-seq) clustering analysis revealed that 6iL-mESCs formed a distinct transcriptional cluster relative to both canonical naïve mESCs (2i/LIF) and primed epiblast stem cells (EpiSCs) cultured in Activin A, FGF2, and XAV939 (AFX) medium (Supplementary information, Fig. S4f). Notably, the expression of the formative-stage regulator *Otx2*^26,27^ in 6iL-mESCs more closely resembled that of EpiSCs and formative stem cells than that of classical naïve mESCs. These data suggest that, consistent with our BRD0705/IWR1 findings,^20^ 6iL/E4 preserves functional naïve-like ESC pluripotency while stabilizing a distinct transcriptional state that supports multiple pluripotent states rather than a conventional ground state.

Functional assays confirmed robust developmental competence. In vitro embryoid body (EB) differentiation generated derivatives of all three germ layers (Fig. 2d), and teratoma formation demonstrated multilineage differentiation in vivo (Fig. 2e). GFP-labeled 6iL-mESCs contributed extensively to chimeric embryos across multiple developmental stages (Fig. 2f; Supplementary information, Fig. S5), with broad distribution across all three germ layers (Supplementary information, Fig. S6). Importantly, GFP^+^ cells were detected in the gonads of E13.5 chimeric embryos and expressed NANOS3, a germ cell-specific marker (Fig. 2g; Supplementary information, Fig. S7a), demonstrating germline contribution. GFP^+^ embryonic germ cell (EGC) lines were derived from chimeric gonads under 2i/LIF conditions, as described previously,^28,29^ and expanded long-term (Supplementary information, Fig. S7b–d). These EGCs expressed TFAP2C and OCT4 (Supplementary information, Fig. S7e, f), supporting germ cell identity and suggesting that 6iL-mESCs retain germ cell differentiation potential in vivo.

We next evaluated rESCs. rESCs initially derived under 2i conditions were passaged in 6iL/E4 for at least 6 passages while maintaining typical ESC morphology. Immunofluorescence analysis showed uniform expression of NANOG and OCT4 (Fig. 2h), indicating preservation of the undifferentiated state. Principal component analysis (PCA) integrating 6iL-rESC transcriptomes with embryonic reference datasets spanning E3.5–E6.5 positioned 6iL-rESCs near early epiblast populations and closely overlapping with conventional 2i-cultured rESCs, while remaining clearly distinct from rat EpiSCs (Fig. 2i). Importantly, when 6iL-cultured rESCs were reverted to standard 2i conditions, they readily adapted and resumed conventional growth (Fig. 2j), indicating that 6iL does not compromise developmental plasticity.

Functional differentiation assays further confirmed the pluripotency of 6iL-rESCs. EB differentiation generated derivatives of all three germ layers (Fig. 2k). Teratomas derived from 6iL-rESCs contained tissues representing all three embryonic germ layers as well as prominent extraembryonic endodermal structures, including yolk sac-like epithelium and giant trophoblast-like cells (Supplementary information, Fig. S8), indicating broad developmental competence. Under germline induction conditions, 6iL-rESCs generated primordial germ cell-like cells (PGC-LCs), as evidenced by co-expression of TFAP2C and PRDM1 (Fig. 2l) and upregulation of additional primordial germ cell (PGC)-associated genes (Fig. 2m).

Collectively, these results demonstrate that 6iL/E4 supports the stable, long-term maintenance of rodent ESCs while preserving genomic stability, pluripotency-associated transcriptional programs, and broad developmental competence. Although definitive germline transmission remains to be established, the robust chimera contribution observed here indicates substantial developmental potential. More broadly, the ability of a single defined culture condition to sustain ESCs across both rodent and non-rodent mammals suggests the existence of a conserved yet flexible pluripotency regulatory framework that can be captured through precise modulation of signaling pathways.

### Generation and characterization of human PSCs in 6iL/E4

**Fig. 3:**
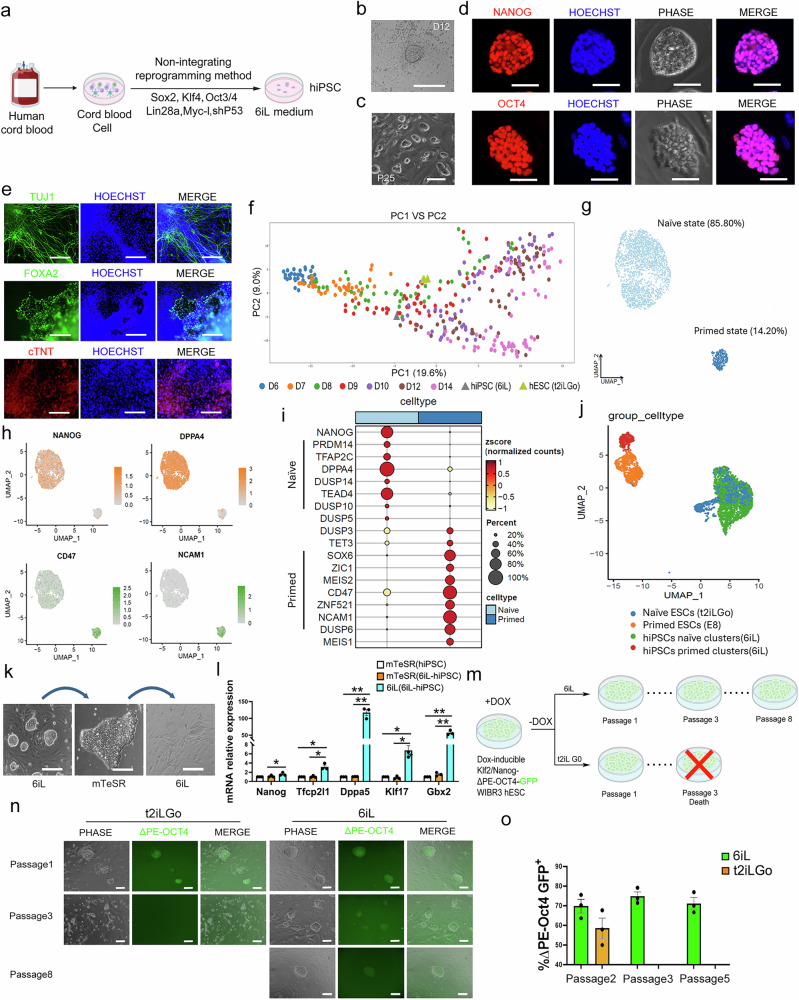
Generation and characterization of human PSCs using 6iL. **a** Schematic of the reprogramming strategy for generating 6iL-hiPSCs (created with BioRender.com). **b** Representative phase-contrast image of a 6iL-hiPSC clone derived from human cord blood cells at day 12 (D12) of reprogramming. Scale bar, 200 µm. **c** Representative images of hiPSCs cultured in 6iL for 25 passages. Scale bar, 100 µm. **d** IF analysis confirming expression of the pluripotency markers NANOG and OCT4 in 6iL-hiPSCs. Hoechst (blue) marks nuclei. Scale bars, 50 µm. **e** Representative IF images of EB outgrowths derived from 6iL-hiPSCs, showing expression of lineage-specific markers: TUJ1 (ectoderm, green), FOXA2 (endoderm, red), and cTNT (mesoderm, red). Scale bars, 200 µm. **f** PCA showing scRNA-seq transcriptional dynamics across differentiation time points (D6–D14). Each dot represents an individual sample colored by developmental stage, with hiPSCs in 6iL for 27 passages and hESCs cultured in t2iLGo (published data) indicated by triangles. Human embryonic development reference data were obtained from GSE136447,^31^ and the t2iLGo reference datasets were derived from ERR590398, ERR590399, and ERR590400.^33^ **g** UMAP visualization of scRNA-seq data from 6iL-hiPSCs (P27) showing segregation into naïve and primed pluripotent states. Each dot represents an individual cell, with 85.80% classified as naïve and 14.20% as primed. **h** UMAP feature plots showing expression of representative naïve pluripotency markers (*NANOG* and *DPPA4*) and primed markers (*CD47* and *NCAM1*) in scRNA-seq data from 6iL-hiPSCs. Color intensity indicates relative gene expression levels across individual cells. **i** Dot plot showing expression of representative naïve and primed pluripotency markers in scRNA-seq data from 6iL-hiPSCs. Dot size indicates the percentage of cells expressing each gene, and color intensity represents scaled expression levels (*Z*-score of normalized counts). **j** Integrated UMAP visualization showing transcriptional relationships between reference naïve ESCs (t2iLGo), primed ESCs (E8), and naïve and primed clusters identified from 6iL-hiPSC scRNA-seq data. t2iLGo and E8 reference data: E-MTAB-6819. **k** Representative phase-contrast images showing morphological transitions of hiPSCs during culture condition switching, including cells maintained in 6iL, transitioned to mTeSR medium for 7 passages, and subsequently reverted to 6iL conditions. Scale bars, 200 µm. **l** qRT-PCR analysis comparing the expression of pluripotency marker genes in 6iL-hiPSCs cultured under 6iL conditions, 6iL-hiPSCs transitioned to mTeSR1 conditions for 3 passages, and the hiPSC line maintained in mTeSR1 medium. Data are presented as mean ± SEM. *P < 0.05, **P < 0.01, ***P < 0.001. **m** Schematic illustrating a comparative assay evaluating the ability of different culture conditions to maintain naïve hESC identity. WIBR3 hESCs carrying DOX-inducible *Klf2/Nanog* and a ΔPE-OCT4-GFP reporter were cultured under either 6iL or t2iLGo conditions following DOX withdrawal. Cells maintained in 6iL continued to propagate over multiple passages, whereas cells cultured in t2iLGo failed to sustain survival and underwent cell death by P3, indicating differential support for naïve hESC maintenance (created with BioRender.com). **n** Representative phase-contrast a**n**d ΔPE-OCT4-GFP fluorescence images comparing maintenance of naïve hESC identity under t2iLGo and 6iL culture conditions following DOX withdrawal in inducible *Klf2/Nanog* WIBR3 hESCs. Scale bars, 100 µm. **o** Quantification of the proportion of ΔPE-OCT4-GFP^+^ cells under 6iL and t2iLGo culture conditions across passages following DOX withdrawal in inducible *Klf2/Nanog* WIBR3 hESCs.

Having shown that 6iL/E4 supports rodent and non-rodent ESCs, we asked whether the defined system could support the establishment and maintenance of human PSCs. Integration-free hiPSCs were generated by plasmid reprogramming of cord blood cells using SOX2, KLF4, OCT4, L-MYC, and LIN28A.^30^ Following electroporation, cells were plated on feeders and switched into 6iL/E4 on day 3 (Fig. 3a). Multiple compact, iPSC-like colonies emerged by day 12 and were isolated for clonal expansion (Fig. 3b). Individual lines expanded stably in 6iL/E4 and tolerated single-cell passaging without the addition of a Rho-associated coiled-coil-containing protein kinase (ROCK) inhibitor (Fig. 3c).

Immunofluorescence analysis confirmed homogeneous expression of NANOG and OCT4 in long-term 6iL-hiPSC cultures (Fig. 3d). Karyotype analysis showed no major chromosomal abnormalities after extended culture (2n = 46, 82.5%, 33/40 metaphases at passage 30; Supplementary information, Fig. S9a). Functionally, 6iL-hiPSCs formed EBs and teratomas containing derivatives of all three germ layers (Fig. 3e; Supplementary information, Fig. S9b). Under PGC induction conditions, 6iL-hiPSCs generated PGC-LCs, demonstrating preserved germline differentiation potential (Supplementary information, Fig. S9c, d).

Transcriptomic profiling placed 6iL-hiPSCs within an early post-implantation developmental window. PCA against human embryonic reference datasets (day 6 (D6)–D14)^31^ positioned 6iL-hiPSCs closest to the D8–D9 developmental interval and showed strong alignment with hESCs cultured under reported naïve conditions (t2iLGo) (Fig. 3f). scRNA-seq analysis of long-term 6iL-hiPSC cultures identified two transcriptional subpopulations: a dominant naïve-like cluster (85.8%) and a smaller primed-like cluster (14.2%) (Fig. 3g). Marker analysis showed enrichment of naïve-associated genes (e.g., *NANOG*, *DPPA4*) in the major cluster and elevated expression of primed markers (e.g., *CD47*, *NCAM1*) in the minor cluster (Fig. 3h, i). Integration with published pluripotent reference datasets^32^ confirmed that the dominant 6iL population closely resembles naïve hESCs maintained in t2iLGo, whereas the minor population maps nearer to conventional primed hESCs in E8 (Fig. 3j; Supplementary information, Fig. S9e–j). Consistent with the single-cell analysis, bulk RNA-seq correlation analysis similarly clustered 6iL-hiPSCs with naïve rather than primed PSCs (Supplementary information, Fig. S9k). Furthermore, quantitative comparison of pluripotency-associated markers revealed elevated expression of naïve-associated genes (*NANOG*, *TEAD4*, and *TFAP2C*) and reduced expression of primed markers (*CD47*, *ZIC2*, and *ZNF521*) in 6iL-hiPSCs relative to cells maintained under primed culture conditions (Supplementary information, Fig. S9l).

Genome-wide DNA methylation profiling by whole-genome bisulfite sequencing (WGBS) revealed that 6iL-hiPSCs exhibit a trend toward hypomethylation relative to primed hiPSCs maintained in conventional mTeSR, a widely used primed medium, broadly mirroring features observed in other naïve culture conditions (Supplementary information, Fig. S10). Although the precise methylation landscape differed from that of the t2iLGo-cultured cells, the overall reduction in global methylation is consistent with a naïve-like epigenetic configuration.

We next compared 6iL-cultured hiPSCs with cells maintained in mTeSR. Transferring 6iL-hiPSCs to mTeSR induced a rapid morphological shift from compact, dome-shaped colonies to flattened, epithelial colonies; conversely, cells maintained in mTeSR showed poor survival and extensive differentiation when transferred back to 6iL (Fig. 3k). qRT-PCR confirmed that 6iL-hiPSCs express higher levels of naïve-associated genes (e.g., *NANOG*, *TFCP2L1*, *DPPA5*, *KLF17*, and *GBX2*) than the same cells after transition to mTeSR or a conventional hiPSC line maintained in mTeSR (Fig. 3l). These data indicate that 6iL/E4 enforces a transcriptionally and functionally distinct naïve-like state that is not readily interchangeable with the primed state sustained by mTeSR.

To test the robustness of 6iL in a resetting paradigm, we used WIBR3 hESCs carrying a ΔPE-OCT4-GFP reporter and a doxycycline (DOX)-inducible KLF2/NANOG cassette.^33^ DOX induction activated ΔPE-OCT4-GFP, indicating resetting (Supplementary information, Fig. S9m); after DOX withdrawal, cells maintained in 6iL persisted for at least 8 passages and retained GFP expression, whereas cells cultured in the previously reported t2iLGo condition^33,34^ progressively died and lost GFP positivity by passage 3 (Fig. 3m–o). Thus, 6iL more effectively preserves reset human pluripotency following transcription factor withdrawal.

In summary, 6iL/E4 enables the generation, long-term expansion, and stable maintenance of human PSCs in a predominantly naïve-like state with preserved multilineage and germline differentiation capacity. Importantly, the use of E4 as a fully defined, serum-free basal medium enhances the translational appeal of the 6iL system for preclinical and clinical applications.

### Hippo pathway modulation enables stable derivation of chimera-competent rabESCs under 6iL/E4

**Fig. 4:**
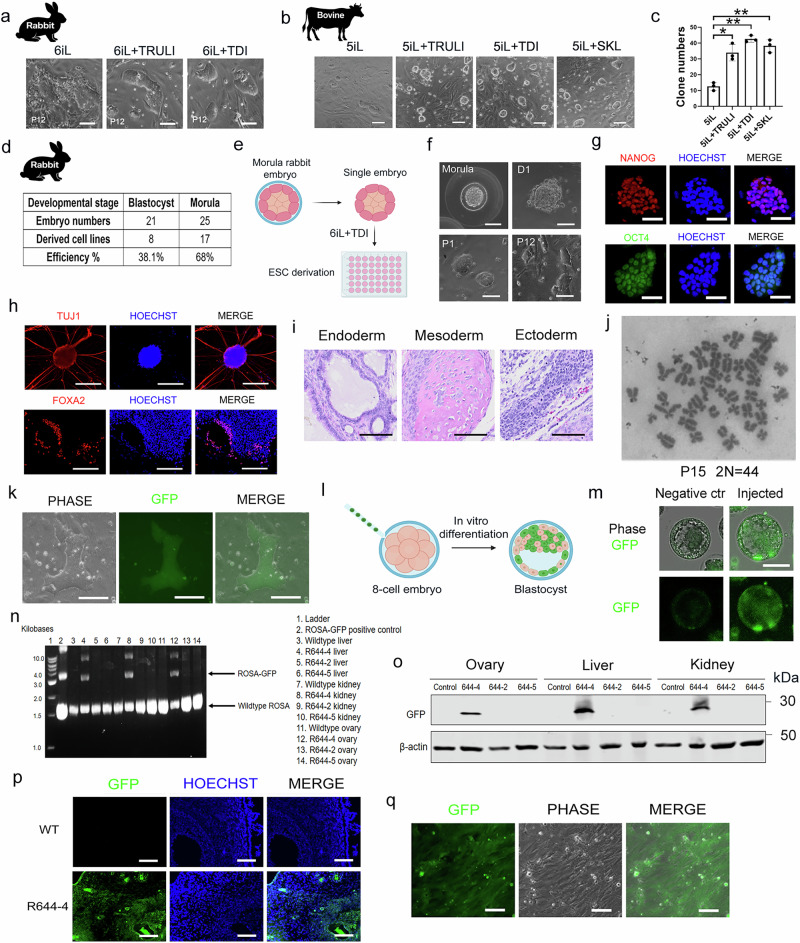
Derivation and characterization of rabESCs cultured in 6iL/TDI. **a** Representative phase-contrast images of rabESCs derived from morula-stage embryos in 6iL, 6iL+TRULI, or 6iL+TDI in E4 medium at P12. Scale bars, 100 µm. **b** Representative phase-contrast images of bESCs cultured under 5iL conditions with additional small molecules (TRULI, TDI, and SKL2001). Scale bars, 200 µm. **c** Quantification of bESC colony numbers under 5iL conditions with the indicated additional small molecules in **b**. Data are presented as mean ± SEM. *P < 0.05, **P < 0.01. **d** Summary table showing the efficiency of rabESC derivation from embryos at different developmental stages in 6iL/TDI. **e** Schematic representation of rabESC derivation from individual morula embryos in 6iL/TDI. **f** Representative phase-contrast images showing rabbit embryo development and ESC derivation in 6iL/TDI, including morula-stage embryos, day 1 (D1) outgrowths, and rabESC colonies at early (P1) and later (P12) passages. Scale bars, 100 µm. **g** Representative IF images showing NANOG and OCT4 expression in rabESCs derived in 6iL/TDI E4. Hoechst (blue) marks nuclei. Scale bars, 50 µm. **h** Representative IF images of EB outgrowths derived from 6iL/TDI-rabESCs showing expression of germ layer markers: TUJ1 (ectoderm, red) and FOXA2 (endoderm, red). Scale bars, 100 µm. **i** Representative H&E staining of teratomas formed by 6iL/TDI-rabESCs (P16), demonstrating differentiation into tissues representing all three embryonic germ layers. Scale bars, 100 µm. **j** Representative karyotype of 6iL/TDI-rabESCs at P15, showing a normal chromosomal complement (2n = 44). **k** Representative phase-contrast and fluorescent images showing GFP-labeled rabESC colonies derived in 6iL/TDI. Scale bars, 100 µm. **l** Schematic illustrating the generation of chimeric rabbit embryos by transferring GFP-labeled 6iL/TDI-rabESCs into 8-cell embryos, which were then cultured in vitro to the blastocyst stage (created with BioRender.com). **m** Representative fluorescence images showing the contribution of GFP^+^ rabESCs to rabbit chimeric embryos. ~10 GFP-labeled rabESCs (P9) were injected into each 8-cell-stage rabbit embryo, and fluorescence images show their successful integration into blastocysts. Scale bar, 100 μm. **n** Genomic DNA PCR results show the presence of GFP alleles in the liver, kidney, and ovary of chimeric rabbits derived from blastocysts injected with GFP-labeled 6iL/TDI-rabESCs. **o** Western blot analysis showing GFP protein expression in the liver, kidney, and ovary of live-born offspring derived from blastocyst transfer following injection of GFP-labeled 6iL/TDI-rabESCs. β-actin was used as a loading control. **p** Representative IF images showing GFP expression in ovarian tissue of the chimeric rabbit (R644-4) derived from GFP-labeled 6iL/TDI-rabESCs. Scale bars, 100 µm. **q** Fluorescence images of ovarian cells isolated and cultured from the R644-4 chimeric rabbit, showing GFP^+^ cells. Scale bars, 100 µm.

To further extend the applicability of 6iL/E4 to non-rodent mammals, we optimized conditions for rabESC derivation. Although 6iL alone enabled initial rabESC establishment, a subset of clones underwent spontaneous differentiation after approximately 10 passages (Fig. 4a), indicating incomplete stabilization of self-renewal.

To enhance long-term maintenance, we screened additional small molecules and identified the LATS1/2 inhibitor TRULI and its optimized derivative TDI-011536 (TDI).^35^ Supplementation with either compound in 6iL markedly improved rabESC propagation (Fig. 4a). Immunofluorescence analysis demonstrated that TDI treatment induced robust nuclear translocation of Yes-associated protein (YAP) in rabbit, human, mouse, and bovine ESCs (Supplementary information, Fig. S11a–d), consistent with inhibition of Hippo signaling and activation of YAP-dependent transcription.

Functionally, TDI supplementation significantly increased AP^+^ rabESC colony formation under 6iL conditions (Supplementary information, Fig. S11e, f). Conversely, disruption of the YAP–TEAD interaction using verteporfin^36^ reduced colony numbers and partially abrogated TDI-mediated enhancement (Supplementary information, Fig. S11e, f), indicating that YAP activation contributes directly to ESC self-renewal in this context. Notably, replacement of SKL2001 with TRULI or TDI in bESC cultures yielded comparable or improved expansion efficiency (Fig. 4b, c), and similar effects were observed in mouse and rat ESCs (data not shown). These findings suggest that modulation of Hippo/YAP signaling is a conserved mechanism that supports pluripotency across species.

rabESCs were efficiently derived under 6iL supplemented with TDI (6iL/TDI), with higher derivation efficiency observed from morula-stage embryos than from blastocyst-stage embryos (Fig. 4d). Under 6iL/TDI conditions, rabESCs sustained long-term expansion (Fig. 4e, f) while maintaining robust expression of NANOG and OCT4 (Fig. 4g). Although TDI markedly improved rabESC propagation, occasional differentiation was still observed in some lines during extended passaging around passage 18 (Supplementary information, Fig. S12g). Pluripotency was validated through functional differentiation assays. EB differentiation generated derivatives of all three germ layers, including ectoderm (Fig. 4h), endoderm (Fig. 4h), and mesoderm (Supplementary information, Video S1). Teratoma analysis further confirmed the presence of tissues representing all three embryonic germ layers (Fig. 4i). Karyotype analysis at passage 15 confirmed maintenance of a normal chromosome complement (2n = 44, 75%, 30/40 metaphases; Fig. 4j), indicating gross chromosomal stability.

To assess developmental competence in vivo, GFP-labeled 6iL/TDI-rabESCs were established (Fig. 4k) and microinjected into 8-cell-stage host embryos from non-GFP rabbits (Fig. 4k, l). Injected embryos efficiently developed to the blastocyst stage, exhibiting a high degree of chimerism (Fig. 4m; Supplementary information, Fig. S12a–f). A total of 139 injected embryos were transferred into six pseudopregnant recipients, yielding 16 live-born kits, of which seven (43%) survived to weaning (6 weeks). PCR genotyping and western blot analysis identified one female animal (R644-4) as GFP^+^, demonstrating ESC contribution (Fig. 4n, o). Immunofluorescence analysis of ovarian tissue from R644-4 revealed GFP^+^ cells (Fig. 4p), providing histological evidence of ESC-derived chimerism. To confirm signal specificity, ovarian cells were isolated and cultured ex vivo; live-cell imaging confirmed persistent GFP expression (Fig. 4q).

As further evidence of genome-editing compatibility, we generated homozygous *Tyr*-edited rabESC lines from single-cell-derived 6iL-rabESCs using CRISPR/Cas9 (Supplementary information, Fig. S12h), supporting the suitability of these cells for precise genetic modification.

Collectively, these results demonstrate that incorporation of Hippo pathway inhibition into 6iL/E4 stabilizes rabESC self-renewal and enables the derivation of chimera-competent rabESCs, establishing 6iL/TDI as a robust platform for rabESC generation.

### Derivation and characterization of 6iL-bESCs

**Fig. 5:**
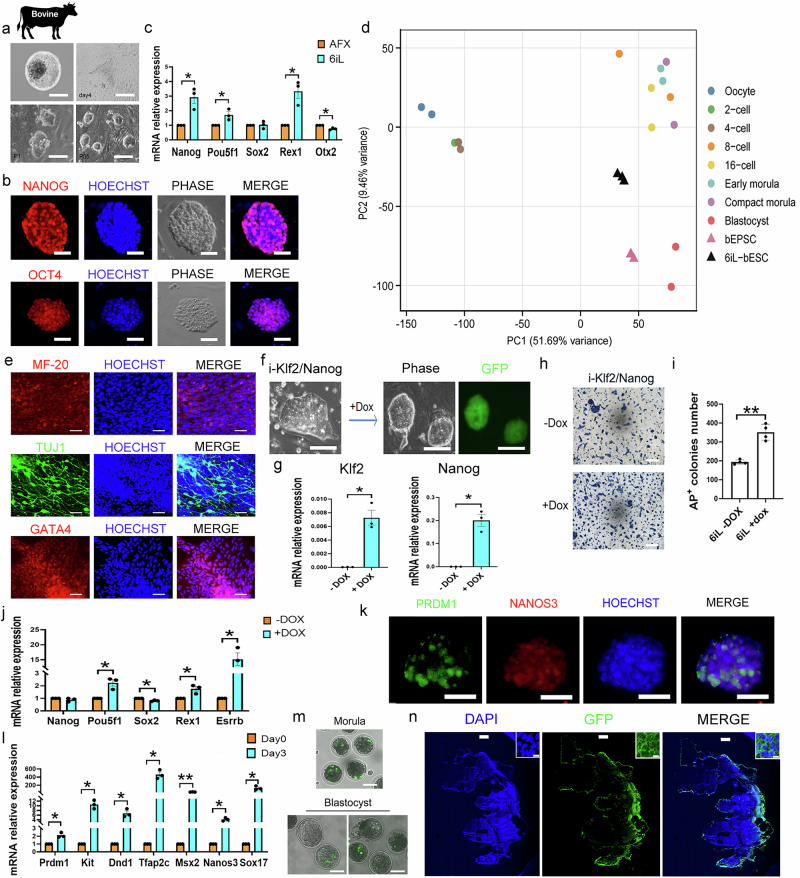
Derivation and characterization of 6iL-bESCs. **a** Representative images of bovine blastocysts, outgrowths, and ESC colonies (P1 and P35) derived in 6iL. Scale bars, 100 µm. **b** Representative IF images of 6iL-bESCs showing NANOG and OCT4 expression. Scale bars, 50 µm. **c** qRT-PCR analysis comparing the expression of pluripotency markers (*Nanog, Pou5f1, Sox2*, and *Rex1*) and the primed marker *Otx2* in 6iL-bESCs vs AFX-cultured cells. Data are presented as mean ± SEM; *P < 0.05. **d** PCA showing the developmental positioning of P28 6iL-bESCs relative to bovine preimplantation embryos and bEPSCs. Transcriptomes of 6iL-bESCs and bEPSCs were compared with bovine embryonic reference datasets spanning the oocyte-to-blastocyst stages, revealing clustering patterns along developmental progression. Reference datasets for bovine embryonic development were obtained from GSE59186, and the bEPSC reference dataset was obtained from GSE129760. **e** Representative IF images of EB outgrowths derived from 6iL-bESCs showing differentiation into the three germ layers: mesoderm (MF-20), ectoderm (TUJ1), and endoderm (GATA4). Scale bars, 50 µm. **f** Representative images of GFP-labeled 6iL-bESCs carrying a DOX-inducible Klf2/Nanog system. Scale bars, 100 µm. **g** qRT-PCR analysis showing DOX-induced Klf2 and Nanog expression in inducible 6iL-bESCs. Gene expression levels are presented as relative mRNA expression (mean ± SEM). *P < 0.05. **h** Representative images of AP-stained inducible Klf2/Nanog 6iL-bESCs cultured in the absence (−DOX) or presence (+DOX) of doxycycline, showing enhanced colony formation upon DOX induction. Scale bars, 200 µm. **i** Quantification of AP^+^ colony numbers under −DOX and +DOX conditions shown in **h**. Data are presented as mean ± SEM. **P < 0.01. **j** qRT-PCR analysis of pluripotency gene expression in inducible Klf2/Nanog 6iL-bESCs cultured in the absence (−DOX) or presence (+DOX) of doxycycline. Data are presented as mean ± SEM. *P < 0.05. **k** Representative IF images of EBs derived from 6iL-bESCs showing the expression of PRDM1 (green) and NANOS3 (red). Scale bars, 50 µm. **l** qRT-PCR of PGC marker genes during differentiation of 6iL-bESCs into PGC-LCs. Data are presented as mean ± SEM; *P < 0.05, **P < 0.01. **m** Representative images of morula- and blastocyst-stage bovine embryos injected with DOX-inducible GFP-labeled 6iL-bESCs. Scale bars, 100 µm. **n** Representative IF images of bovine embryo sections from E40 chimeras, which developed from blastocyst-stage embryos injected with DOX-inducible Klf2/Nanog-expressing GFP-labeled bESCs. Scale bars, 1000 μm. Insets show magnified views. Scale bars, 10 μm.

We next investigated whether 6iL/E4 could support the derivation and long-term maintenance of ESCs from large mammals, using cattle as a representative model. The 6iL condition efficiently enabled the derivation of bESCs from bovine blastocysts (Fig. 5a). The resulting lines maintained stable, dome-shaped colony morphology for more than 35 passages in vitro (Fig. 5a) and robustly expressed core pluripotency markers, including NANOG and OCT4 (Fig. 5b).

A previous study reported bovine embryonic disc stem cells derived under AFX.^37^ Compared with AFX-maintained cells, 6iL-bESCs exhibited significantly higher expression of pluripotency-associated genes, including *Nanog*, *Pou5f1*, *Klf2*, *Klf4*, *Sall4*, *Stat3*, and *Rex1*, and markedly reduced expression of *Otx2* (Fig. 5c; Supplementary information, Fig. S13a). PCA performed by integrating bovine embryonic reference datasets^38^ positioned 6iL-bESCs near early blastocyst-stage embryos (Fig. 5d). Notably, 6iL-bESCs localized closer to the blastocyst developmental trajectory than previously reported bovine EPSCs (bEPSCs),^38^ suggesting that they correspond to a pluripotent state associated with early blastocyst development.

Functional assays confirmed their multilineage differentiation potential. 6iL-bESCs generated derivatives of all three germ layers in vitro and formed teratomas containing tissues representative of ectoderm, mesoderm, and endoderm in vivo (Fig. 5e; Supplementary information, Fig. S13b). Karyotype analysis at passage 30 demonstrated maintenance of a normal chromosomal complement (2n = 60, 72.5%, 29/40 metaphases at passage 30; Supplementary information, Fig. S13i), indicating genomic stability during prolonged culture.

### Inducible Klf2/Nanog expression reinforces pluripotency and enhances developmental competence of 6iL-bESCs

Recent work has shown that modulation of key pluripotency regulators enhances chimera-forming competency in rabbit iPSCs.^16^ Given the extended reproductive cycle and high economic cost associated with large-animal experiments, we sought to improve the efficiency of bovine chimera formation by reinforcing the pluripotency network in 6iL-bESCs. NANOG sustains OCT4 expression and maintains ESC identity independently of STAT3 signaling,^39^ while KLF2 is negatively regulated by MEK/ERK signaling and promotes naïve pluripotency upon overexpression.^40^ Moreover, transient co-expression of KLF2 and NANOG is sufficient to reset primed human PSCs to a naïve pluripotent state.^33^ We therefore introduced a DOX-inducible Klf2/Nanog cassette into GFP-labeled 6iL-bESCs.

DOX induction led to increased colony compaction (Fig. 5f, g), enhanced proliferative capacity (Fig. 5h, i), and upregulation of pluripotency-associated genes, including *Pou5f1*, *Rex1*, and *Esrrb* (Fig. 5j), indicating reinforcement of the ESC transcriptional network. We next assessed whether 6iL-bESCs retained germline differentiation competence. Following DOX withdrawal, cells were briefly primed with Activin A and bFGF overnight and subsequently induced to differentiate into PGC-LCs using previously established protocols.^41^ Successful differentiation was confirmed by the presence of PRDM1/NANOS3 double-positive cells (Fig. 5k) and the upregulation of PGC-associated genes (Fig. 5l). These results indicate that transient exogenous induction of Klf2/Nanog does not compromise subsequent lineage specification and that 6iL-bESCs retain the capacity to generate PGC-LCs in vitro after withdrawal of the reinforcing factors.

We next assessed their in vivo developmental competence. GFP-labeled 6iL-bESCs carrying the DOX-inducible Klf2/Nanog cassette were microinjected into 8-cell-stage, morula-stage, or early blastocyst-stage bovine embryos (Fig. 5m; Supplementary information, Fig. S13c–e). Injected embryos were cultured to the blastocyst stage to evaluate donor cell contribution. GFP^+^ blastocysts were obtained from 68% (102/150) of morula-injected embryos and 63% (95/150) of blastocyst-injected embryos. Injection into 8-cell embryos yielded a comparable rate of GFP^+^ blastocysts (50%, 6/12), indicating developmental compatibility across stages (Fig. 5m; Supplementary information, Fig. S13c–e).

Blastocysts exhibiting robust GFP signals were transferred into ten recipient cows, resulting in eight confirmed pregnancies. Analysis of E40 conceptuses identified one chimeric embryo with ~18.2% GFP^+^ contribution (Fig. 5n). GFP^+^ donor cells were detected in mesodermal and endodermal tissues (Supplementary information, Fig. S13f), demonstrating in vivo developmental integration of 6iL-bESC derivatives.

To assess genome-editing compatibility, we generated *Prnp*-edited bESC lines from single-cell-derived 6iL-bESCs using CRISPR/Cas9 (Supplementary information, Fig. S13h), demonstrating that these cells are readily amenable to precise genetic modification.

Collectively, these findings demonstrate that 6iL/E4 enables efficient derivation, stable long-term expansion, and functional developmental integration of bESCs. Reinforcement of the pluripotency network through inducible Klf2/Nanog expression enhances colony stability and supports chimera formation without compromising differentiation potential. Beyond its practical utility for large-animal genetic engineering, this work provides conceptual evidence that the core regulatory circuitry governing ESC pluripotency is conserved across mammals, despite substantial differences in phylogeny, embryogenesis, and body size.

## Discussion

We present a defined, serum-free 6iL/E4 culture platform that enables the derivation and long-term propagation of ESCs from multiple mammalian species (mouse, rat, rabbit, and cow) and supports naïve-like human PSCs. By systematically deconstructing basal medium composition and reconstructing signaling inputs, we demonstrate that a shared, tunable signaling architecture can stabilize pluripotency across diverse mammals. These findings support the concept that the core regulatory logic underlying ESC self-renewal is broadly conserved, even though pathway outputs and quantitative requirements differ among species.

Reduction of N2B27 to a minimal four-component basal formulation (E4) eliminated differentiation-promoting activities and provided a clean platform for cross-species interrogation. Importantly, the principal value of E4 appears to lie in this discovery context rather than in an absolute requirement once the optimized signaling environment has been established. Consistent with the comparable performance of E4 and N2B27 under the conventional naïve rodent ESC conditions shown in Supplementary information, Fig. S2, our experience indicates that complete N2B27 can also substitute for E4 within the 6iL framework across multiple species. We therefore view E4 as a defined and experimentally advantageous basal medium that facilitated identification of the 6iL signaling combination while also offering practical advantages in compositional simplicity, storage stability, and mechanistic interpretability. Building upon this foundation, we identified a modular signaling cocktail, 6iL (BRD0705, IWR1, CP67, GDC0879, SKL2001, 828, and LIF), that, with limited species-specific adjustments, supports ESC derivation from rabbit and bovine embryos and sustains rodent ESCs as well as naïve-like human PSCs. The modular structure of 6iL/E4 is conceptually important: rather than requiring fundamentally distinct species-specific pathways, pluripotency can be maintained by balancing conserved signaling inputs, including WNT modulation, selective GSK3α inhibition, suppression of PDGFR and MEK/ERK signaling, and STAT3 activation.

A central insight of this study is that selective inhibition of GSK3α is critical for cross-species ESC derivation. Although pan-GSK3α/β inhibition by CHIR promotes rodent ESC self-renewal through stabilization of β-catenin,^3,7,42^ it induced substantial differentiation in rabbit and bovine cultures (Fig. 1a). In contrast, selective GSK3α inhibition by BRD0705, together with tankyrase inhibition by IWR1, established a permissive but non-differentiating signaling environment.^20^ This balanced state appears to support intrinsic self-renewal pathways while preventing excessive β-catenin transcriptional activation. Although SKL2001 has been reported to function as a WNT activator, our finding that it promotes ESC self-renewal independently of β-catenin (Supplementary information, Fig. S3h), together with our recent study demonstrating that SKL2001 enhances granulocyte-monocyte progenitor (GMP) expansion through a WNT/β-catenin-independent mechanism,^43^ suggests that SKL2001 may exert broader context-dependent activities beyond canonical WNT signaling.

We further identified PDGFR inhibition as a critical component of efficient ESC derivation across species. CP67 consistently suppressed non-epiblast outgrowths and enabled stable colony formation, suggesting that inhibition of primitive endoderm (PrE) differentiation is essential for capturing epiblast-derived ESCs. This interpretation is supported by developmental studies demonstrating prominent PDGFR expression within PrE lineages.^44–47^ Although additional mechanistic studies are required, our findings indicate that limiting PDGF signaling helps prevent early lineage divergence and facilitates the stabilization of pluripotency during ESC derivation.

Our results indicate that activation of the STAT3 signaling pathway is important for the maintenance of ESCs across species, consistent with its well-established role in rodent ESCs. Previous work reported rabbit pluripotent stem cell lines maintained by bFGF and Activin/Nodal signaling without exogenous LIF,^48^ but chimera-forming capacity was not demonstrated in that study, making direct comparison with the chimera-contributing rabbit cells derived here under 6iL/TDI difficult. We therefore interpreted the apparent differences in dependence on LIF as likely reflecting the stabilization of distinct rabbit pluripotent states, rather than a direct contradiction. Notably, the 6iL/TDI-rabESCs established in this study exhibited chimera-forming ability, further supporting our conclusion that these cells represent ESC state. Likewise, the finding that 6iL-bESCs correspond to an early embryonic developmental stage provides additional support for this conclusion.

Modulation of the Hippo/YAP pathway emerged as another conserved axis supporting ESC propagation under defined culture conditions. In our system, LATS1/2 inhibition by TDI or TRULI enhanced rabbit and bovine ESC self-renewal, accompanied by YAP nuclear localization and increased colony formation; furthermore, addition of TDI or TRULI to the 6iL condition improved ESC expansion across mouse, rat, rabbit, human, and bovine ESCs. However, the role of Hippo/YAP signaling in pluripotency appears highly context-dependent. Although previous studies have suggested that YAP promotes ESC stemness^49^ and naïve reprogramming,^50^ recent evidence in human naïve PSCs indicates that YAP activation may be incompatible with naïve pluripotency.^51^ These apparently divergent findings suggest that YAP does not exert a universal effect across all pluripotent states or culture systems, but instead acts in a manner dependent on the broader signaling environment, including MEK/ERK, WNT, GSK3α, PDGFR, and STAT3. Thus, we do not interpret our findings as evidence that YAP activation universally promotes naïve pluripotency; rather, our results suggest that Hippo pathway modulation can enhance ESC propagation under the specific signaling context established by 6iL. Notably, TDI or TRULI could each functionally substitute for SKL2001 in supporting bESC self-renewal, and supplementation with SKL2001, TDI, or both, together with a core module composed of IWR1, BRD0705, CP67, GDC0879, LIF, and 828, supported efficient ESC expansion across multiple mammalian species. The precise mechanisms by which TDI/TRULI promotes ESC propagation, as well as the functional distinctions and long-term consequences of ESCs maintained under these related conditions, remain to be fully elucidated.

Species-specific developmental dynamics likely contribute to the long-standing difficulty of deriving naïve ESCs from non-rodent mammals. In mice, embryonic diapause extends the window during which naïve epiblast cells can be captured, whereas higher mammals undergo continuous preimplantation development without a comparable arrest phase.^52–54^ This distinction may partially explain why conventional 2i conditions fail in many non-rodent species. Our results suggest that careful tuning of conserved signaling pathways — and, in the case of bovine ESCs, transient reinforcement of core pluripotency regulators such as Klf2 and Nanog — can overcome some of these constraints. Importantly, transient reinforcement did not compromise subsequent differentiation capacity, as cells retained germline differentiation potential and the ability to integrate during in vivo development.

Although the regulation of pluripotency exhibits clear species-specific features, the six small molecules in the 6iL condition mainly target core developmental signaling pathways that are broadly conserved across mammals, including WNT, GSK3α, FGF/MEK, Hippo/YAP, and STAT3 signaling networks. These pathways play fundamental roles during early embryogenesis and have repeatedly been implicated in the control of cell fate, proliferation, and pluripotency maintenance in mouse, rat, rabbit, bovine, and human embryos. Thus, our findings do not imply that pluripotency is regulated identically across species. Rather, they suggest that the underlying signaling logic is conserved, whereas species-dependent differences arise from variation in pathway context, feedback strength, and regulatory network architecture. In this framework, conserved signaling modules are deployed in distinct quantitative and transcriptional contexts in different species. Consistent with this model, comparative transcriptomic analyses in our study showed that ESCs established under 6iL conditions across multiple species converged on comparable developmental-stage transcriptional programs despite their distinct regulatory landscapes. This cross-species functional convergence suggests that modulation of conserved signaling nodes is sufficient to stabilize analogous pluripotent states across mammals. Thus, the broad applicability of the 6iL system likely reflects its targeting of evolutionarily conserved developmental signaling hubs, whereas species-specific phenotypic outcomes reflect differences in regulatory feedback and cellular context rather than fundamental divergence in the core pathways themselves.

Several limitations should be acknowledged. Although 6iL/E4 enabled the derivation of rabbit and bovine ESCs and yielded chimeric contribution, germline transmission remains to be established and requires further optimization and larger-scale validation. Additionally, some rabESC lines exhibited gradual differentiation during extended passaging (Supplementary information, Fig. S12g), indicating that further refinement will be necessary to achieve fully stable long-term maintenance. Moreover, although 6iL/E4 supports functionally pluripotent states across species, the resulting cells often occupy transcriptionally distinguishable, developmentally poised configurations rather than being exact equivalents of mouse ground-state naïve ESCs. Accordingly, the precise developmental identity of rabESCs should be considered provisional until higher-quality, stage-resolved rabbit embryonic reference datasets become available. In addition, although Klf2/Nanog reinforcement enabled functional assessment of bESC developmental potential in the current study, developing a bovine ESC culture system without genetic reinforcement by exogenous Klf2/Nanog remains an important future goal. Finally, the molecular mechanisms underlying the noncanonical effects of compounds such as SKL2001 remain to be elucidated.

Despite these challenges, the 6iL/E4 system provides a broadly applicable, serum-free platform for cross-species pluripotency research. Beyond its practical value in large-animal genetic engineering, disease modeling, and conservation biology, this work offers conceptual evidence that mammalian pluripotency is governed by a conserved yet tunable signaling architecture. By integrating minimal basal conditions with mechanism-informed pathway modulation, we establish a framework for extending authentic ESC derivation to additional mammalian species. Continued mechanistic dissection and functional validation will further refine this platform and deepen our understanding of conserved pluripotency networks across evolution.

## Materials and methods

### Ethics statement

The human ESC/iPSC experiments in this study were reviewed and approved by the University of Southern California Stem Cell Research Oversight Committee under Protocol No. 2023-2 and were conducted in accordance with applicable institutional guidelines and regulations. This study used previously established hESCs, and no human participants, human specimens, or identifiable human data were newly recruited or collected; therefore, no additional informed consent was required.

### Mice

Adult female mice were used in this study. Timed-pregnant B6D2F1 mice were used for cell line derivation, C57BL/6J mice were used as blastocyst donors for chimera generation, and ARC mice served as embryo transfer recipients. ESC injection and blastocyst transfer were performed at the Irvine Transgenic Mouse Facility (University of California, Irvine; Institutional Animal Care and Use Committees (IACUC) protocol #AUP-22-126) and the Transgenic Animal Model Core at the University of Michigan. Mouse embryos for mESC derivation were collected at the Department of Animal Resources, University of Southern California (IACUC protocol # 20230). All animal procedures were conducted in accordance with institutional guidelines and were approved by the IACUC of the respective institutions.

### Bovine

Non-lactating, 3-year-old crossbreed (*Bos taurus* × *Bos indicus*) cows were used as recipients for chimera experiments. The animal experiments were conducted under animal use protocols (202300000191) approved by the IACUC of the University of Florida. All cows were housed in open pasture and under the constant care of the farm staff.

### Rabbit

New Zealand White (NZW) rabbits were used in this study. The animal maintenance, care, and use procedures were reviewed and approved by the IACUC (protocol #PRO00011844) of the University of Michigan. All procedures were carried out in accordance with the approved guidelines.

### Harvesting mouse embryos

Female B6D2F1 mice (8–10 weeks old) were superovulated by intraperitoneal injection of 5 IU pregnant mare serum gonadotropin (PMSG; Prospec, Rehovot, Israel), followed 48 h later by intraperitoneal injection of 5 IU human chorionic gonadotropin (hCG). Females were then mated with C57BL/6 males, and the presence of a vaginal plug the following morning was designated as E0.5. Blastocysts were flushed from the uterine horns at E3.5.

### Bovine in vitro embryo production

Germinal vesicle (GV)-stage oocytes were collected as cumulus–oocyte complexes (COCs), aspirated from slaughterhouse ovaries. In vitro maturation was performed in BO-IVM medium (IVF Bioscience, Falmouth, UK) at 38.5 °C with 6% CO_2_ for 22–23 h to obtain metaphase II (MII) oocytes. Cryopreserved semen from a Holstein bull with proven fertility was prepared in BO-SemenPrep medium (IVF Bioscience) and added to drops containing COCs at a final concentration of 2 × 10⁶ spermatozoa/mL for in vitro fertilization (IVF). Gametes were co-incubated at 38.5 °C and 6% CO_2_. After 10 h (for microinjection experiments) or 16 h (for non-microinjection experiments) in BO-IVF medium (IVF Bioscience), IVF embryos were denuded of cumulus cells by vortexing for 5 min in BO-Wash medium (IVF Bioscience) and then cultured to the blastocyst stage on day 7.5 in BO-IVC medium (IVF Bioscience) at 38.5 °C, 6% CO_2_, and 6% O_2_. Embryos at various developmental stages were evaluated under light microscopy according to the International Embryo Technology Society’s grading standards.

### Rabbit embryo collection and culture

Superovulation, embryo collection, and culture were conducted as previously described.^55^ Briefly, adult NZW female rabbits were superovulated with follicle-stimulating hormone (FSH; Folltropin-V; Bioniche Life Sciences, Belleville, Canada) and hCG (Chorulon; Intervet, Millsboro, DE, USA) to induce ovulation, followed by breeding with male rabbits. Eighteen hours post-breeding, the zygote-stage embryos were collected and cultured in embryo culture medium in vitro to the blastocyst stage. The embryo culture medium was composed of 10% FBS (10438-026; Thermo Fisher, Waltham, MA, USA), MEM non-essential amino acids (M7145; Thermo Fisher), BME amino acid solution (B6766; MilliporeSigma, Burlington, MA, USA), 2 mM L-glutamine (25030081; Thermo Fisher), and 0.4 mM sodium pyruvate (11360070; Thermo Fisher) in Earle’s Balanced Salts (E2888; MilliporeSigma).

### Derivation and culture of 6iL-mESCs

E3.5 mouse blastocysts were briefly exposed to acidic Tyrode’s solution to remove the zona pellucida. The de-zonated embryos were then plated onto feeders prepared from mitotically inactivated MEFs in 6iL medium to derive mESCs (E4 medium supplemented with human LIF (20 ng/mL; PeproTech, Rocky Hill, NJ, USA), IWR1 (2.5 µM; Selleck, Houston, TX, USA), BRD0705 (8 µM; Cayman, Ann Arbor, MI, USA), CP67 (1 µM; Cayman), GDC0879 (1 µM; Selleck), 828 (5 µM; WuXi AppTec, Shanghai, China), and SKL2001 (10 µM; Selleck)). Additionally, Go6983 (1 µM; Selleck) can be selectively added during culture to promote more compact stem cell colonies. After 4–6 days of culture, blastocyst outgrowths were dissociated using 0.025% trypsin and transferred onto freshly prepared feeders for further cultivation. The cells were cultured at 37 °C under 5% CO_2_. E4 medium is a 1:1 mixture of DMEM/F12 and Neurobasal medium, supplemented with insulin (4 μg/mL; Sigma, St. Louis, MO, USA), human holo-transferrin (22 μg/mL; Sigma), BSA (1 mg/mL; Sigma), sodium selenite (12.5 ng/mL; Sigma), and L-glutamine (2 mM; Thermo Fisher).

### Derivation and culture of 6iL-rabESCs

Zona pellucida-removed rabbit morula embryos were placed on feeders and initially cultured in E4 medium supplemented with human LIF (20 ng/mL; PeproTech), IWR1 (2.5 µM; Selleck), BRD0705 (8 µM; Cayman), CP67 (1 µM; Cayman), GDC0879 (1 µM; Selleck), 828 (5 µM; WuXi AppTec), SKL2001 (10 µM; Selleck), and TDI-011536 (100 nM; Selleck) for 4–6 days. The outgrowths were then dissociated using 0.025% trypsin and transferred onto freshly prepared feeders for further cultivation. The cells were maintained at 38.5 °C under 5% CO_2_.

### Derivation and culture of 6iL-bESCs

ICMs isolated from bovine blastocysts were placed on feeders and initially cultured in E4 medium (with an additional 50 µg/mL bovine transferrin (T1283; Sigma)) supplemented with bovine LIF (20 ng/mL; Kingfisher Biotech, St. Paul, MN, USA), IWR1 (2.5 µM; Selleck), BRD0705 (8 µM; Cayman), CP67 (1 µM; Cayman), GDC0879 (1 µM; Selleck), 828 (5 µM; WuXi AppTec), and SKL2001 (10 µM; Selleck) for 4–6 days. To enhance cell expansion, the culture medium was additionally supplemented with TRULI (2 µM; Selleck) or TDI-011536 (100 nM; Selleck). Outgrowths were then dissociated using 0.025% trypsin and transferred onto freshly prepared feeders for further expansion. Individual bESC clones were picked and dissociated for passaging. Cells were maintained at 38.5 °C under 5% CO_2_.

### Derivation and culture of human iPSCs using 6iL

Human umbilical cord blood cells were collected and electroporated with the plasmids pCXLE-hOCT3/4-shp53, pCXLE-hSK, and pCXLE-hUL.^30^ After culture in StemSpan™ SFEM II medium (STEMCELL Technologies, Vancouver, Canada) for 2 days, the medium was replaced with E4 medium supplemented with human LIF (20 ng/mL; PeproTech), IWR1 (2.5 µM; Selleck), BRD0705 (8 µM; Cayman), CP67 (1 µM; Cayman), GDC0879 (1 µM; Selleck), 828 (5 µM; WuXi AppTec), and SKL2001 (10 µM; Selleck). Cells were cultured on feeders. Go6983 (1 µM; Selleck) was optionally added during culture to promote more compact colony morphology. After approximately 10–12 days, individual colonies were picked and passaged onto feeder-coated plates using 0.025% trypsin digestion. Cells were maintained at 37 °C under 5% CO_2_.

### 6iL-rESC culture

The DAC8 rat ESC line was maintained on feeders in E4 medium supplemented with 6iL. Cultures were incubated at 37 °C with 5% CO_2_, and the medium was changed every 2–3 days. For passaging, cells were dissociated into single cells using 0.025% trypsin.

### 6iL-naïve human ESC culture

OCT4-ΔPE-GFP-WIBR3 hESCs were infected with lentiviruses (FUW-tetO-lox-hKLF2, FUW-tetO-lox-hNANOG, and M2rtTA). The cells were cultured on feeders in E4 medium supplemented with CHIR/PD03/LIF, with DOX added for selection over 3 passages. GFP^+^ individual colonies were then picked and cultured on feeders in the presence of human LIF (20 ng/mL; PeproTech), IWR1 (2.5 µM; Selleck), BRD0705 (8 µM; Cayman), CP67 (1 µM; Cayman), GDC0879 (1 µM; Selleck), 828 (5 µM; WuXi AppTec), and SKL2001 (10 µM; Selleck). Additionally, Go6983 (1 µM; Selleck) was optionally added during culture to improve cell conditions. Cultures were incubated at 37 °C with 5% CO_2_, and the medium was changed every 2–3 days. For passaging, cells were dissociated into single cells using 0.025% trypsin.

### Mouse chimera generation and histological analysis

Approximately 10–15 dissociated cells were introduced into individual blastocyst-stage embryos by microinjection. The injected embryos were subsequently transferred into pseudopregnant foster females for further development. Chimeric embryos collected between E6.5 and E13.5 were imaged using a BZ-X800 fluorescence microscope (Keyence, Itasca, IL, USA). For PGC analysis, chimeric embryos were recovered at E13.5. Chimeric embryos were subsequently processed for paraffin sectioning and cryosection-based staining, whereas dissected gonads were processed for cryosectioning and immunostaining. For cryosections, embryos or dissected gonads were fixed in 4% paraformaldehyde (PFA) at 4 °C for 2–4 h (or overnight for larger embryos), followed by cryoprotection in 15% sucrose and then 30% sucrose in PBS until the tissues sank. Samples were embedded in OCT compound and frozen on dry ice. Cryosections were cut at a thickness of 8–10 μm using a Leica cryostat (Wetzlar, Germany). For paraffin sectioning, embryos were fixed in 10% neutral buffered formalin at room temperature overnight, followed by dehydration through a graded ethanol series, clearing in xylene, and embedding in paraffin. Paraffin blocks were sectioned to a thickness of 5 μm using a microtome and mounted onto glass slides.

### Generation and culture of DOX-inducible Klf2/Nanog overexpressing bESCs

OCT4-ΔPE-GFP-WIBR3 bESCs were transduced with lentiviral vectors carrying DOX-inducible expression constructs for human KLF2 and NANOG. The lentiviral constructs used were FUW-tetO-lox-hKLF2 (Addgene, Watertown, MA, USA), FUW-tetO-lox-hNANOG (Addgene), and the reverse tetracycline transactivator FUW-M2rtTA (Addgene). Lentiviral production and infection were performed following standard protocols. Following infection, cells were plated onto feeder layers and cultured in E4 medium supplemented with 6iL and PD0325901 (100 nM; Selleck). To induce transgene expression, DOX (2 μg/mL; Sigma) was added to the culture medium. Cells were maintained under these conditions for 5 passages to allow stable establishment of the inducible overexpression system. After the selection period, the resulting inducible cell lines were maintained in 6iL/E4 medium supplemented with DOX, which sustained overexpression of KLF2 and NANOG.

### AP staining

The AP substrate solution (Vector Laboratories, Newark, CA, USA) was prepared following the manufacturer’s instructions. Cells were incubated with the AP substrate at room temperature for 20–30 min in the dark. After incubation, they were fixed with 4% (w/v) PFA at room temperature for 1 h.

### Quantitative real-time PCR (qRT-PCR) analysis

Total RNA was extracted using an RNeasy Mini Kit (Qiagen, Hilden, Germany) according to the manufacturer’s protocol. cDNA was synthesized using an iScript cDNA Synthesis Kit (Bio-Rad, Hercules, CA, USA). qRT-PCR was performed with iTaq Universal SYBR® Green Supermix (Bio-Rad) on a ViiA 7 real-time PCR system. Gene expression levels were normalized to *Gapdh*.

### Immunofluorescence

Cells were fixed in a 4% PFA solution for 15 min at room temperature, followed by three PBS washes. Next, the cells were blocked with 5% BSA in PBS containing 0.3% Triton X-100 for 1 h. Primary and secondary antibodies — diluted in 1% BSA in PBS with 0.3% Triton X-100 — were subsequently applied for either 1 h at room temperature or overnight at 4 °C. Details of the antibodies used can be found in the Key Resources Table.

### EB formation and differentiation

EBs were formed using AggreWell 400 plates (STEMCELL Technologies) in accordance with the manufacturer’s protocol. EBs from different species were cultured for 2–4 days. For cardiomyocyte differentiation, the resulting EBs were plated onto gelatin-coated dishes and cultured in either IMDM/10% FBS or GMEM/10% FBS medium. For neural differentiation, EBs were plated onto gelatin-coated dishes and maintained in N2B27 medium.

### Primordial germ cell-like cell induction

EBs were formed using AggreWell 400 plates (STEMCELL Technologies). The basic steps for PGC-LC induction and the culture medium recipe were carried out according to a published protocol.^41,56,57^ Rat and bovine ESCs were first cultured overnight in N2B27 medium containing Activin A (20 ng/mL; STEMCELL Technologies) and bFGF (20 ng/mL; PeproTech). 6iL-hiPSCs were directly placed into PGC induction medium. EBs were formed in PGC-LC induction medium: GK15 medium (GMEM, KnockOut serum replacement (15% (v/v); Thermo Fisher), NEAA (0.1 mM; Thermo Fisher), sodium pyruvate (0.1 mM; Thermo Fisher), β-mercaptoethanol (0.1 mM; Sigma), and GlutaMAX (2 mM; Thermo Fisher)) supplemented with BMP4 (200 ng/mL; Gibco, Grand Island, NY, USA), LIF (1000 U/mL; PeproTech), SCF (100 ng/mL; R&D Systems, Minneapolis, MN, USA), and EGF (50 ng/mL; PeproTech).

### Lentiviral infection

Lentiviruses pseudotyped with vesicular stomatitis virus glycoprotein G (VSV-G) and packaged using psPAX2 were produced in HEK-293 cells following established protocols. In brief, the culture medium was replaced 12 h after transfection, and the virus-containing supernatant was harvested between 48 and 72 h post-transfection. The collected supernatant was then filtered through a 0.45 µm filter. Finally, the viral supernatants (FUW-tetO-loxP-hKLF2, FUW-tetO-loxP-hNANOG, and FUW-M2rtTA) were added to the 6iL-bESCs or hESCs. Two rounds of infection were carried out over a 24 h period, each in the presence of 2 μg/mL polybrene.

### Whole-genome bisulfite sequencing

Genomic DNA samples were processed at the University of Michigan Epigenomics Core for enzymatic methyl-seq (EM-seq) library construction. DNA concentration was determined using a Qubit dsDNA BR Assay Kit (Thermo Fisher), and sample integrity was evaluated with the Agilent 4200 TapeStation Genomic DNA assay (Agilent Technologies, Santa Clara, CA, USA). For library preparation, 100 ng of genomic DNA per sample was used as input for the NEBNext Enzymatic Methyl-seq kit (NEB, Ipswich, MA, USA), according to the manufacturer’s protocol. Libraries were amplified by PCR for 5 cycles using NEBNext Multiplex Oligos designed for EM-seq applications. Following amplification, library concentrations were measured using a Qubit dsDNA HS Assay Kit (Thermo Fisher), and fragment size distribution was examined on an Agilent 4200 TapeStation with the High Sensitivity D1000 ScreenTape. Library quantification was further validated by qPCR using the KAPA Illumina Library Quantification Kit (KAPA Biosystems, Wilmington, MA, USA). Equimolar amounts of individual libraries were pooled, re-quantified by qPCR, and subjected to paired-end 150 bp sequencing on the NovaSeq X Plus platform (Illumina, San Diego, CA, USA) using a 10B 300-cycle shared flow cell at the Advanced Genomics Core, University of Michigan.

### scRNA-seq library preparation and data analysis

Library preparation was performed using a Single Cell 3′ RNA Prep, T2 kit (Illumina). For indexing, the Mix 7 index set was used for 6iL-hiPSC, and the Mix 8 index set was used for 6iL-mESC. Next-generation sequencing was performed on a NovaSeq X Plus (Illumina), with 10B 2 × 150 200 M PE (100 M each direction) reads per sample.

To analyze single-cell particle-templated instant partitioning sequencing (PIP-seq) data, FASTQ files were aligned to the human or mouse reference genome downloaded from the pipseeker database (GRCh38.p13 (GENCODE v40 2022.04, Ensembl 106) or GRCm39 (GENCODE vM29 2022.04, Ensembl 106)) using pipseeker in full mode with the following options: --chemistry V, --fastq, --remove-bam, --star-index-path, --output-path, --max-sensitivity 5. Count matrices from sensitivity_3 were selected for the downstream analysis. The generated count matrices from all samples were integrated using the R package Seurat (v4.3.0) and canonical correlation analysis (CCA) with default parameters.^58^ The data were scaled for linear and non-linear dimensionality reduction using PCA and UMAP, respectively. Subsequent clustering and visualization were performed using the standard Seurat workflow with the parameters “dim = 1:30” in the “FindNeighbors” function and “resolution = 0.2” in the “FindClusters” function. The function “FindAllMarkers” in Seurat was used to identify differentially expressed genes (DEGs) in each defined cluster. DEGs were defined using a cutoff of *P*.adjust < 0.05, and fold change > 0.25. The UMAP plots and bubble plots with marker genes were generated using “CellDimPlot” and “GroupHeatmap” functions in R package SCP (v0.4.0) (https://github.com/zhanghao-njmu/SCP), respectively. Gene ontology (GO) and pathway analysis were performed using the R package clusterProfiler (v4.6.1), and GO terms were presented using the “dotplot” function in Seurat.

For 6iL-hiPSC PCA analysis, PCA was performed on log2-transformed (FPKM + 1) gene expression data from human embryo samples (GSE136447). Marker genes representing ICM, pre-epiblast, post-epiblast, and PSA-EPI lineages were obtained from a published method,^44^ and the union of these genes was used for analysis. Samples from developmental days D6–D14 were selected, and the expression matrix was transposed so that samples were treated as observations. PCA was conducted using scikit-learn, and the first two principal components were computed. For integration analyses, scRNA-seq data from 6iL were aggregated into a pseudo-bulk profile and combined with naïve ESC bulk RNA-seq datasets treated with t2iLGo medium (ENA: E-MTAB-5674) using shared genes. All samples were standardized (*Z*-score across genes), PCA was fit on embryo samples, and external samples were projected onto the same PCA space for visualization.

### Bulk RNA-seq library preparation and data analysis

Total RNA from individual cell lines was extracted using an RNeasy Micro Kit (Qiagen). RNA-seq libraries for 6iL-mESCs, 6iL-hiPSCs, and 6iL-bESCs were generated using a SMART-Seq2 v4 kit (Takara Bio, Mountain View, CA, USA) and Nextera XT DNA Library Preparation Kit (Illumina) and multiplexed by Nextera XT Indexes (Illumina) following the manufacturer’s instructions. The concentrations of sequencing libraries were determined using a Qubit dsDNA HS Assay Kit (Life Technologies, Carlsbad, CA, USA) and a KAPA Library Quantification Kit (KAPA Biosystems). The sizes of sequencing libraries were determined using the Agilent D5000 ScreenTape with TapeStation 4200 system (Agilent Technologies). Pooled indexed libraries were then sequenced on the Illumina NovaSeq platform with 150 bp paired-end reads. Bulk RNA-seq libraries from 6iL-rESCs, 2iL-mESCs (N2B27/E4), and 2i-rESCs (N2B27/E4) were prepared and sequenced at the Advanced Genomics Core, University of Michigan. The pooled libraries were subjected to paired-end sequencing on the NovaSeq X Plus platform (System Suite v1.3.1.59007; Illumina) according to the manufacturer’s instructions. BCL Convert Conversion Software (v4.3.13; Illumina) was used to generate demultiplexed FASTQ files.

For 6iL-mESCs, FASTQ files for public RNA-seq were downloaded from GEO (GSE131553). For public RNA-seq and RNA-seq generated in this study, read counts were obtained using Salmon (v0.14.1) and GENCODE (vM24). DEGs were identified using DESeq2 (v1.40.2), requiring a false discovery rate (FDR) of < 0.05 and an absolute fold change ≥ 1.5. For the heatmaps, TPM-normalized counts were quantile normalized using the “normalize.quantiles” function from the “preprocessCore” R package, and then they were *Z*-score normalized by gene. The heatmaps were generated using the “heatmap.2” function from the “gplots” R package, using the “hclust” function with the “ward.D2” method for clustering.

For 6iL-hiPSCs and 6iL-bESCs, multiplexed sequencing reads that passed filters were trimmed to remove low-quality reads and adapters by Trim Galore (v0.6.7) (-q 25 –length 20 –max_n 3 –stringency 3). The quality of reads after filtering was assessed by FastQC, followed by alignment to the human or bovine genome (GRCh38 or ARS-UCD1.3) by HISAT2 (v2.2.1) with default parameters. The output SAM files were converted to BAM files and sorted using SAMtools (v1.14). Read counts of all samples were quantified using featureCounts (v2.0.1) with the reference genome. PCA and cluster analysis were performed with R (a free software environment for statistical computing and graphics). DEGs were identified using edgeR in R. DEGs were defined by an FDR < 0.05 and a fold change > 2. GO and Kyoto Encyclopedia of Genes and Genomes (KEGG) pathway analyses were performed using clusterProfiler in R.

Transcriptome analyses of 6iL-rESCs, 2iL-mESCs (N2B27/E4), and 2i-rESCs (N2B27/E4) were performed in R. Raw data processing, annotation, and gene expression matrix harmonization were carried out at the Advanced Genomics Core, University of Michigan, where duplicated genes were averaged based on gene symbols and normalized expression values were log-transformed. PCA was performed using embryo transcriptomes as the reference dataset, and external samples were projected into the same PCA space using the corresponding reference parameters. DEGs were visualized by hierarchical clustering heatmaps based on row-scaled expression values.

### Bovine in vivo chimera assay

Approximately 10 GFP^+^ bESCs were injected gently into the morula or early blastocyst-stage bovine embryos using a piezo-assisted micromanipulator attached to an inverted microscope (Olympus, Tokyo, Japan). The injected embryos were cultured in a 75:25 mixture of BO-IVC medium and bESC culture medium at 38.5 °C, 6% CO_2_, and 6% O_2_ for an additional 8 h. The injected blastocysts with clear GFP fluorescence were then transferred into non-lactating, 3-year-old crossbreed recipient cows (*n* = 10). Recipient cows were synchronized with a standard 7-day controlled internal drug release (CIDR; Zoetis, Parsippany, NJ, USA) protocol, followed by one intramuscular (IM) dose of ovulation-inducing gonadotropin-releasing hormone (Fertagyl; Merck Animal Health, Rahway, NJ, USA). On day 7, CIDR was removed, and one dose of prostaglandin (Lutalyse; Zoetis) was administered. On day 9, one dose of Fertagyl was administered to stimulate ovulation. Embryo transfer was performed 7 days later. On day 30 after transplantation, pregnancy was diagnosed by ultrasonography. The recipient cows were slaughtered at the University of Florida Meat Processing Center, and the reproductive tracts were harvested to collect day 40 fetuses for analysis of chimeric competence.

### CRISPR/Cas9-mediated gene editing of 6iL-ESCs

6iL-bESCs or 6iL-rabESCs were electroporated with Cas9 ribonucleoprotein complexes containing HiFi Cas9 Nuclease protein (2000 ng; IDT, Coralville, IA, USA) and GenCRISPR/Cas9™ SafeEdit sgRNA (100 pmol; GenScript, Piscataway, NJ, USA) using the Neon Transfection System (Thermo Fisher). For each reaction, Cas9 protein and sgRNA were preassembled in Buffer R at room temperature for 20 min. The resulting mixture was then combined with 1 × 10^5^ ESCs in a total volume of 10 μL. Electroporation was performed using a 10 μL Neon tip with the following program: 1400 V, 20 ms, 1 pulse. After electroporation, cells were directly transferred onto feeder-coated culture plates in 6iL or 6iL/TDI medium, and the medium was changed the next day. Once individual colonies appeared, single clones were picked and transferred into 96-well plates containing feeders in 6iL or 6iL/TDI medium, with one clone per well. The clones were subsequently expanded by passaging, and genomic DNA was analyzed to assess genome-editing efficiency.

### Cryosectioning and immunofluorescence analysis of bovine fetuses

For immunofluorescence staining of bovine chimeric fetal cryosections, fetuses were collected from the uterus and washed three times with PBS, after which they were immersed in 4% PFA overnight at 4 °C. After washing with PBS, they were sequentially dehydrated in 10%, 20%, and 30% sucrose, followed by OCT:30% sucrose (1:1), with each dehydration step performed for 4 h. Next, fetuses were embedded in Tissue-Plus O.C.T. Compound (4585; Thermo Fisher) and held on dry ice for quick freezing. The frozen OCT blocks were sectioned at 10 μm using a CryoStar NX50 (Thermo Fisher). Sections were permeabilized with 1% Triton X-100 in PBS for 30 min, rinsed with wash buffer, and then blocked in blocking buffer (0.1% Triton X-100, 1% BSA, 0.1 M glycine, and 10% donkey serum) for 2 h at room temperature. Subsequently, the sections were incubated overnight at 4 °C with an anti-GFP primary antibody (600-101-215; Rockland, Pottstown, PA, USA). Following primary antibody incubation, sections were incubated with Fluor 488-conjugated secondary antibodies for 1 h at room temperature and then stained with DAPI (D1306; Invitrogen, Carlsbad, CA, USA) for 15 min. Images were captured with a fluorescence confocal microscope (Leica).

### Rabbit embryonic stem cell injection and embryo transfer

ESC injection into 8-cell-stage rabbit embryos was carried out as previously described.^55^ Briefly, rabESCs were trypsinized to single cells before injection. Ten ESCs were injected into each 8-cell-stage rabbit embryo. The ESC-injected embryos were either cultured in vitro to evaluate the ESC contribution to the ICM in blastocyst-stage embryos or transferred to pseudopregnant recipient animals.

### Teratoma formation

A total of 2 × 10⁶ cells were suspended in 50 µL of 6iL/E4 medium supplemented with Matrigel and injected subcutaneously into 10-week-old immunodeficient NOD-SCID mice. Teratomas were harvested 6–8 weeks post-injection and fixed in 10% neutral buffered formalin. The samples were subsequently paraffin-embedded, sectioned, and subjected to H&E staining for histological analysis.

### Karyotyping

ESCs were treated with 100 ng/mL colcemid (15212012; Thermo Fisher) for 2 h to arrest the cell cycle at metaphase and then trypsinized into single cells. Cells were then treated with 0.075 M potassium chloride (10575090; Thermo Fisher) for 6 min and fixed with 25% acetic acid (320099; MilliporeSigma) in methanol (34860; MilliporeSigma) for 10 min at room temperature. Following three washes with 25% acetic acid in methanol, cells were dropped on a glass slide to form chromosome spreads, and the spreads were stained with Giemsa stain (10092-013; Thermo Fisher). Chromosome spreads were randomly selected and imaged under the microscope (BZ800; Keyence), and chromosome numbers were manually counted.

### RNA-seq

Multiplexed sequencing reads that passed filters were trimmed to remove low-quality reads and adapters by Trim Galore (v0.6.7). The quality of reads after filtering was assessed using FastQC, followed by alignment to the bovine genome (ARS-UCD1.3) using HISAT2 (v2.2.1) with default parameters. The output SAM files were converted to BAM files and sorted using SAMtools (v1.14). Read counts of all samples were quantified using featureCounts (v2.0.1) with the reference genome. PCA and cluster analysis were performed with R. DEGs were identified using edgeR in R and defined by an FDR < 0.05 and a fold change > 2. GO and KEGG pathway analyses were performed using clusterProfiler in R.

### Statistical analysis

All data are presented as means ± SEM. Experiments were repeated at least three times. Student’s *t*-test (two-tailed) was used to evaluate statistical significance, and error bars represent the SEM of three independent experiments. *P* < 0.05 was taken to indicate statistical significance, which is indicated as follows: *P < 0.05, **P < 0.01, and ***P < 0.001.

## Supplementary information

Supplementary information, Fig. S1

Supplementary information, Fig. S2

Supplementary information, Fig. S3

Supplementary information, Fig. S4

Supplementary information, Fig. S5

Supplementary information, Fig. S6

Supplementary information, Fig. S7

Supplementary information, Fig. S8

Supplementary information, Fig. S9

Supplementary information, Fig. S10

Supplementary information, Fig. S11

Supplementary information, Fig. S12

Supplementary information, Fig. S13

Supplementary information, Table S1

Supplementary information, Table S2

Supplementary information, Table S3

Supplementary information, Table S4

Supplementary information, Table S5

Supplementary information, Table S6

Supplementary information, Table S7

Supplementary information, Table S8

Supplementary information, Table S9

Supplementary information, Video legend

Supplementary information, Video S1

## Data Availability

All study data are included in the article and/or supplementary information. The bulk RNA-seq data have been deposited in GEO (GSE295390 and GSE295412) and are also provided in Supplementary information, Tables S6–S9. The scRNA-seq data have been deposited in GEO (GSE325170). The WGBS data have been deposited in GEO (GSE333356).
