# Supplementary material for: A universal 6iL/E4 culture system for deriving and maintaining embryonic stem cells across mammalian species
Source: Cell Res. 2026 Jul 13;36(8):611–28. doi: 10.1038/s41422-026-01276-y (PMC13424318; doi:10.1038/s41422-026-01276-y)
Supplement: Supplementary file 1 — Supplementary information, Fig. S1 [file 41422_2026_1276_MOESM1_ESM.pdf]

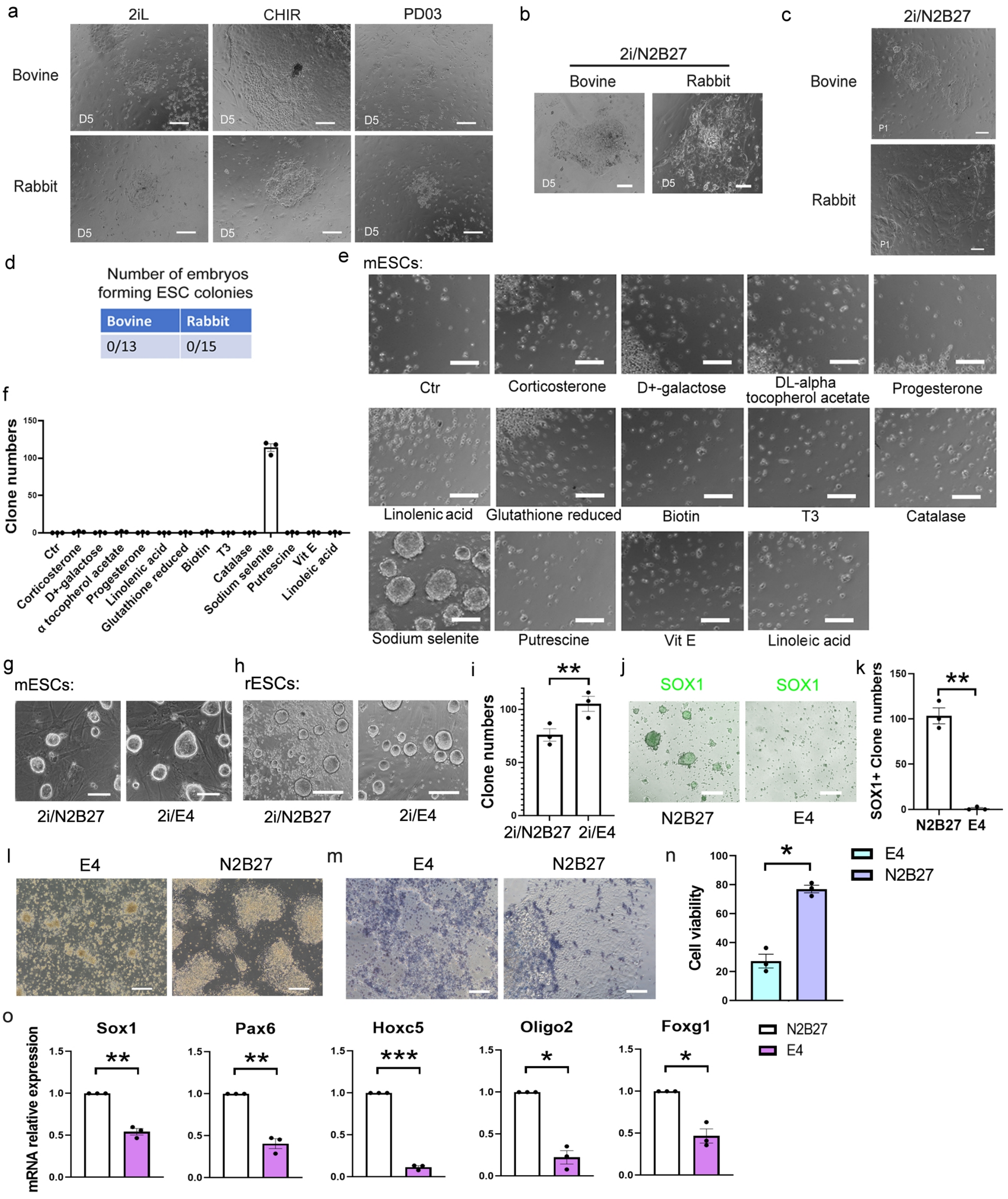
a
2iL
CHIR
PD03
Bovine
Rabbit
D5
b
2i/N2B27
Bovine
Rabbit
D5
c
2i/N2B27
Bovine
Rabbit
P1
d
Number of embryos forming ESC colonies
Bovine
Rabbit
0/13
0/15
e
mESCs:
Ctr
Corticosterone
D+-galactose
DL-alpha tocopherol acetate
Progesterone
Linolenic acid
Glutathione reduced
Biotin
T3
Catalase
Sodium selenite
Putrescine
Vit E
Linoleic acid
f
Clone numbers
150
100
50
0
Ctr
Corticosterone
D+-galactose
α tocopherol acetate
Progesterone
Linolenic acid
Glutathione reduced
Biotin
T3
Catalase
Sodium selenite
Putrescine
Vit E
Linoleic acid
g
mESCs:
2i/N2B27
2i/E4
h
rESCs:
2i/N2B27
2i/E4
i
Clone numbers
**
100
50
0
2i/N2B27
2i/E4
j
SOX1
SOX1
N2B27
E4
k
SOX1+ Clone numbers
**
150
100
50
0
N2B27
E4
l
E4
N2B27
m
E4
N2B27
n
Cell viability
*
100
80
60
40
20
0
E4
N2B27
o
mRNA relative expression
Sox1
Pax6
Hoxc5
Oligo2
Foxg1
**
**
***
*
*
1.5
1.0
0.5
0.0
N2B27
E4

Fig. S1 Optimization of N2B27 composition to improve ESC self-renewal.

a Representative phase-contrast images of primary embryos cultured under different conditions. Bovine ICMs and rabbit morulae were cultured for 5 days (D5) in 2iL, CHIR, or PD03 conditions in N2B27 medium. Scale bars, 200 μm.

b Phase-contrast images of bovine and rabbit blastocysts cultured in 2i/N2B27 for 5 days.

c Representative morphology of bovine and rabbit embryo outgrowths after the first passage (P1) under the 2i condition, showing loss of typical ESC colony morphology. Scale bars, 200 μm.

d Quantification of embryos forming ESC colonies under the 2i condition. No stable ESC colonies were obtained from either bovine (0/13) or rabbit (0/15) embryos.

e Phase-contrast images of B6D2F1 mESCs cultured for 3 passages on 0.1% gelatin-coated plates in DMEM-F12/Neurobasal medium supplemented with 2i, insulin (4 μg/mL), transferrin (22 μg/mL), BSA (1000 μg/mL) and the indicated individual B27 components. Scale bar, 200 μm.

f Bar graph showing the numbers of colonies in (**e**).

g Representative phase-contrast images of mESCs cultured for 4 passages in N2B27 or E4 medium supplemented with 2i. Scale bar, 100 μm.

h Representative phase-contrast images of rESCs cultured for 4 passages in 2i/N2B27 or 2i/E4. Scale bar, 200 μm.

i Bar graph showing the numbers of colonies in (**h**). Data are presented as mean ± SEM. **, $p<0.01$.

j Representative fluorescence images of Sox1-GFP^{+} neural stem cells derived from mESCs cultured alone in N2B27 or E4 medium on day 5 of differentiation. Scale bar = 200 μm.

k Quantification of SOX1^{+} colony numbers corresponding to the images in (**j**). Data are presented as mean ± SEM. **, $p<0.01$.

l Representative cell morphology of mESCs differentiated for 6 days in E4 or N2B27. In contrast to N2B27, embryoid body (EB) death was observed under E4 conditions.

m Representative trypan blue staining indicating cell viability of mouse ESCs differentiated for 6 days in E4 or N2B27.

n Quantification of cell viability in (**m**). Bar graph showing the cell viability of EBs at day 6 of differentiation under E4 or N2B27 conditions. Data are presented as mean ± SEM. *, $p<0.05$.

o Bar graph showing differential mRNA expression levels of neural development–associated marker genes in mESCs at day 5 of differentiation under N2B27 or E4 conditions. Data are presented as mean ± SEM. *, $p<0.05$; **, $p<0.01$; ***, $p<0.001$.
