# Supplementary material for: A universal 6iL/E4 culture system for deriving and maintaining embryonic stem cells across mammalian species
Source: Cell Res. 2026 Jul 13;36(8):611–28. doi: 10.1038/s41422-026-01276-y (PMC13424318; doi:10.1038/s41422-026-01276-y)
Supplement: Supplementary file 2 — Supplementary information, Fig. S2 [file 41422_2026_1276_MOESM2_ESM.pdf]

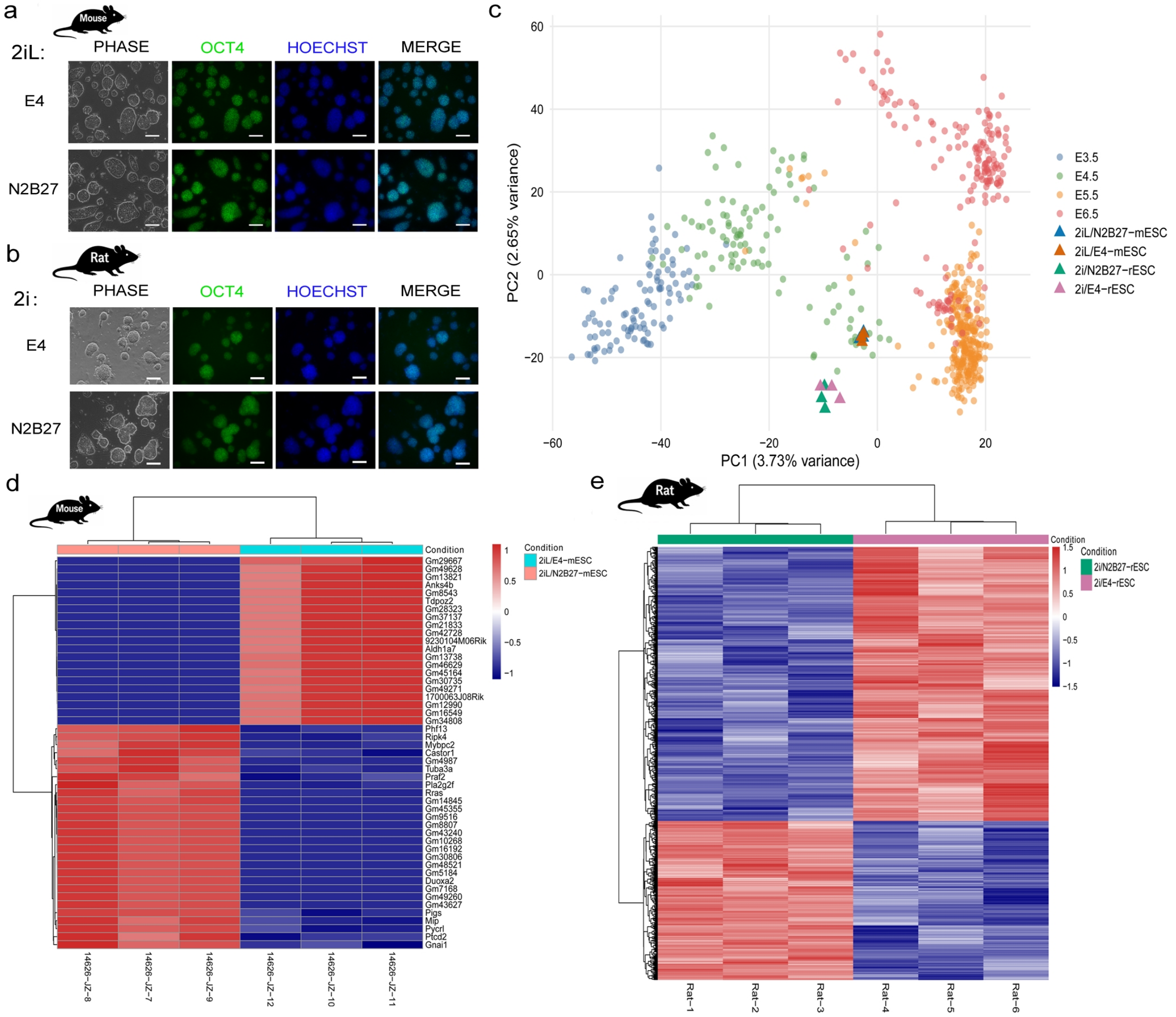

a
Mouse
2iL:
PHASE
OCT4
HOECHST
MERGE
E4
N2B27
b
Rat
2i:
PHASE
OCT4
HOECHST
MERGE
E4
N2B27
c
PC2 (2.65% variance)
PC1 (3.73% variance)
60
40
20
0
−20
−60
−40
−20
0
20
E3.5
E4.5
E5.5
E6.5
2iL/N2B27−mESC
2iL/E4−mESC
2i/N2B27−rESC
2i/E4−rESC
d
Mouse
Condition
2iL/E4−mESC
2iL/N2B27−mESC
1
0.5
0
−0.5
−1
Gm29667
Gm49628
Gm13821
Anks4b
Gm8543
Tdpoz2
Gm28323
Gm37137
Gm21833
Gm42728
9230104M06Rik
Aldh1a7
Gm13738
Gm46629
Gm45164
Gm30735
Gm49271
1700063J08Rik
Gm12990
Gm16549
Gm34808
Phf13
Ripk4
Mybpc2
Castor1
Gm4987
Tuba3a
Praf2
Pla2g2f
Rras
Gm14845
Gm45355
Gm9516
Gm6807
Gm43240
Gm10268
Gm16192
Gm30806
Gm48521
Gm5184
Duoxa2
Gm7168
Gm49260
Gm43627
Pigs
Mip
Pycrl
Pfcd2
Gnai1
14626−JZ−8
14626−JZ−7
14626−JZ−9
14626−JZ−12
14626−JZ−10
14626−JZ−11
e
Rat
Condition
2i/N2B27−rESC
2i/E4−rESC
1.5
1
0.5
0
−0.5
−1
−1.5
Rat−1
Rat−2
Rat−3
Rat−4
Rat−5
Rat−6

Fig. S2 Comparable pluripotent states of mouse and rat ESCs cultured in either E4 or N2B27.

a Representative phase-contrast and immunofluorescence images of mESCs cultured in 2iL in E4 or N2B27 medium for 20 passages. Cells were immunostained for OCT4 (green) and counterstained with Hoechst (blue). Scale bars, 100 μm.

b Representative phase-contrast and immunofluorescence images of rESCs cultured in 2iL in E4 or N2B27 medium for 20 passages. Scale bars, 100 μm.

c PCA of bulk RNA-seq datasets comparing mESCs cultured in 2iL for 15 passages and rESCs in 2i for 22 passages in either N2B27 or E4 medium, together with reference mouse embryonic stages (E3.5, E4.5, E5.5, and E6.5). Samples segregate along principal components according to transcriptional similarity and developmental stage. Reference datasets for mouse E3.5–E6.5 embryos were obtained from GSE100597.

d Hierarchical clustering heatmap showing differentially expressed genes in mESCs cultured in 2iL in E4 versus N2B27 medium, based on bulk RNA-seq analysis. Rows represent genes and columns represent biological replicates (n = 3). Expression values are shown as Z-score–normalized levels.

e Hierarchical clustering of bulk RNA-seq profiles from rESCs cultured in 2i in E4 or N2B27 medium. Rows correspond to genes and columns to biological replicates (n = 3). Expression values are shown as Z-score–normalized levels.
