# Supplementary material for: A universal 6iL/E4 culture system for deriving and maintaining embryonic stem cells across mammalian species
Source: Cell Res. 2026 Jul 13;36(8):611–28. doi: 10.1038/s41422-026-01276-y (PMC13424318; doi:10.1038/s41422-026-01276-y)
Supplement: Supplementary file 3 — Supplementary information, Fig. S3 [file 41422_2026_1276_MOESM3_ESM.pdf]

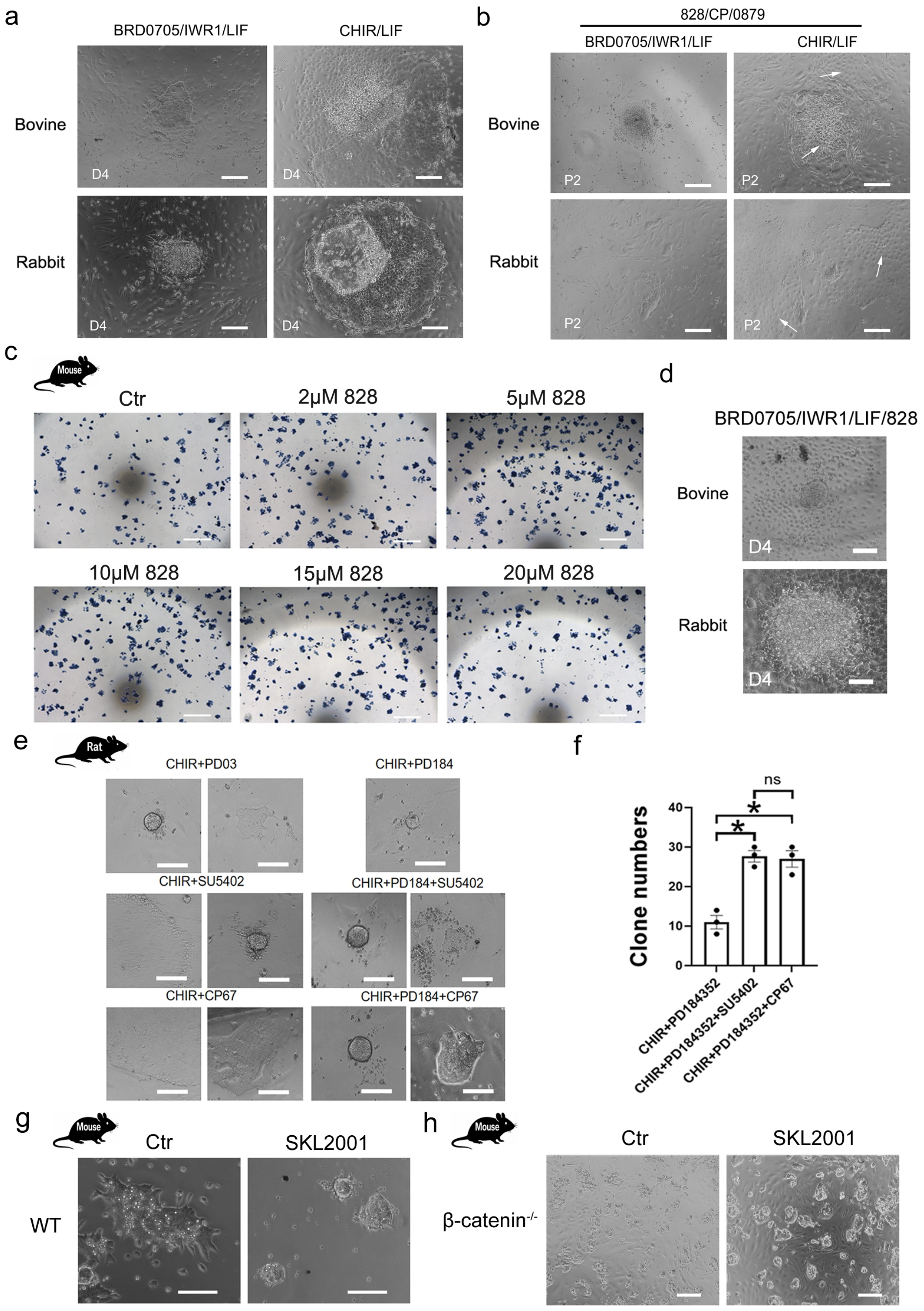
a
BRD0705/IWR1/LIF
CHIR/LIF
Bovine
Rabbit
D4
b
828/CP/0879
BRD0705/IWR1/LIF
CHIR/LIF
Bovine
Rabbit
P2
c
Mouse
Ctr
2μM 828
5μM 828
10μM 828
15μM 828
20μM 828
d
BRD0705/IWR1/LIF/828
Bovine
Rabbit
D4
e
Rat
CHIR+PD03
CHIR+PD184
CHIR+SU5402
CHIR+PD184+SU5402
CHIR+CP67
CHIR+PD184+CP67
f
Clone numbers
ns
*
*
CHIR+PD184352
CHIR+PD184352+SU5402
CHIR+PD184352+CP67
g
Mouse
Ctr
SKL2001
WT
h
Mouse
Ctr
SKL2001
β-catenin-/-

Fig. S3 Optimization of small-molecule conditions for ESC derivation and maintenance across species.

a Representative phase-contrast images of bovine and rabbit ICM outgrowths cultured under BRD0705/IWR1/LIF or CHIR/LIF conditions on day 4. BRD0705/IWR1/LIF supported compact ICM colony formation, whereas CHIR/LIF treatment resulted in enhanced outgrowth expansion accompanied by differentiation of ICM cells. Scale bars, 200 μm.

b Representative phase-contrast images of rabbit and bovine ESC cultures derived from morula-stage (rabbit) and blastocyst-stage (bovine) embryos and maintained under BRD0705/IWR1/LIF or CHIR/LIF conditions in the presence of 828, CP67, and GDC0879 (828/CP/0879) for two passages. Arrows indicate differentiated cells. Scale bars, 200 μm.

c AP staining of colonies formed by mESCs treated with different concentrations of 828 (0, 2, 5, 10, 15, and 20 μM). Representative images are shown. Scale bars, 200 μm.

d Representative phase-contrast images showing the morphology of bovine and rabbit ICMs cultured for 4 days in E4 medium supplemented with BRD0705, IWR1, LIF, and 828. Scale bars, 100 μm.

e Representative phase-contrast images of rabESCs cultured with CHIR in combination with the indicated inhibitors. Scale bars, 100 μm.

f Quantification of colony numbers in (**e**). Data are presented as mean ± SEM. Statistical significance is indicated (*P < 0.05, ns = not significant).

g Representative phase-contrast images of WT mESCs cultured in LIF/serum under control conditions or in the presence of SKL2001. Scale bars, 100 μm.

h Phase-contrast images showing β-catenin$^{-/-}$ mESCs maintained in LIF/serum medium under control conditions or following SKL2001 treatment. Scale bars, 200 μm.
