# Supplementary material for: A universal 6iL/E4 culture system for deriving and maintaining embryonic stem cells across mammalian species
Source: Cell Res. 2026 Jul 13;36(8):611–28. doi: 10.1038/s41422-026-01276-y (PMC13424318; doi:10.1038/s41422-026-01276-y)
Supplement: Supplementary file 4 — Supplementary information, Fig. S4 [file 41422_2026_1276_MOESM4_ESM.pdf]

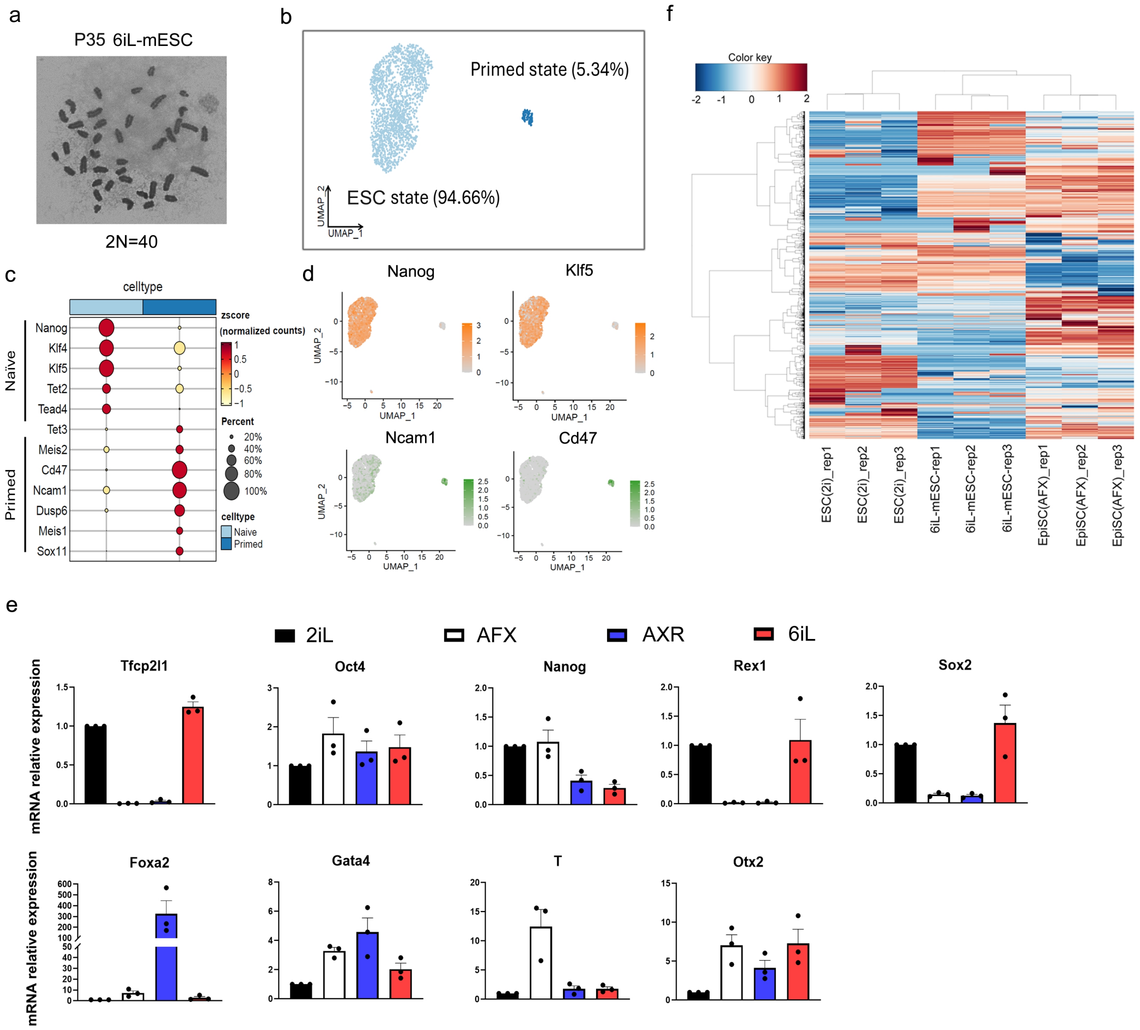
a
P35 6iL-mESC
2N=40
b
Primed state (5.34%)
ESC state (94.66%)
UMAP_2
UMAP_1
c
celltype
Naive
Primed
Nanog
Klf4
Klf5
Tet2
Tead4
Tet3
Meis2
Cd47
Ncam1
Dusp6
Meis1
Sox11
zscore
(normalized counts)
Percent
20%
40%
60%
80%
100%
d
Nanog
Klf5
Ncam1
Cd47
f
Color key
ESC(2i)_rep1
ESC(2i)_rep2
ESC(2i)_rep3
6iL-mESC-rep1
6iL-mESC-rep2
6iL-mESC-rep3
EpiSC(AFX)_rep1
EpiSC(AFX)_rep2
EpiSC(AFX)_rep3
e
2iL
AFX
AXR
6iL
mRNA relative expression
Tfcp2l1
Oct4
Nanog
Rex1
Sox2
Foxa2
Gata4
T
Otx2

Fig. S4 Pluripotency characterization of mESCs derived in 6iL.

a Representative karyotype analysis of P35 6iL-mESCs showing normal chromosomal integrity (2n = 40).

b UMAP projection of single-cell transcriptomic profiles illustrating the distribution of pluripotent states in 6iL-mESCs. The majority of cells cluster within the ESC state (94.66%), whereas a small fraction exhibits a primed-like transcriptional signature (5.34%).

c Dot plot showing the expression patterns of representative naïve and primed pluripotency markers across the identified cell states in 6iL-mESCs. Dot size indicates the percentage of expressing cells, whereas color intensity represents normalized expression levels (z-score).

d Feature plots displaying the expression of representative naïve markers (*Nanog* and *Klf5*) and primed-associated markers (*Ncam1* and *Cd47*) across the UMAP embedding.

e qRT–PCR analysis of pluripotency-associated genes (*Tfcp2l1*, *Oct4*, *Nanog*, *Rex1*, and *Sox2*) and lineage-associated markers (*Foxa2*, *Gata4*, *T*, and *Otx2*) in cells cultured under different conditions: 2iL (naïve ESCs), AFX (primed EpiSCs), AXR (formative stem cells), and 6iL. Data are presented as mean ± SEM. from three independent experiments.

f Hierarchical clustering heatmap of bulk RNA-seq–derived global gene expression profiles comparing ESCs cultured under different conditions. Samples cluster according to pluripotent state and culture condition, revealing distinct transcriptional differences among conventional ESCs, 6iL-mESCs, and epiblast stem cell–like populations.
