# Supplementary material for: A universal 6iL/E4 culture system for deriving and maintaining embryonic stem cells across mammalian species
Source: Cell Res. 2026 Jul 13;36(8):611–28. doi: 10.1038/s41422-026-01276-y (PMC13424318; doi:10.1038/s41422-026-01276-y)
Supplement: Supplementary file 5 — Supplementary information, Fig. S5 [file 41422_2026_1276_MOESM5_ESM.pdf]

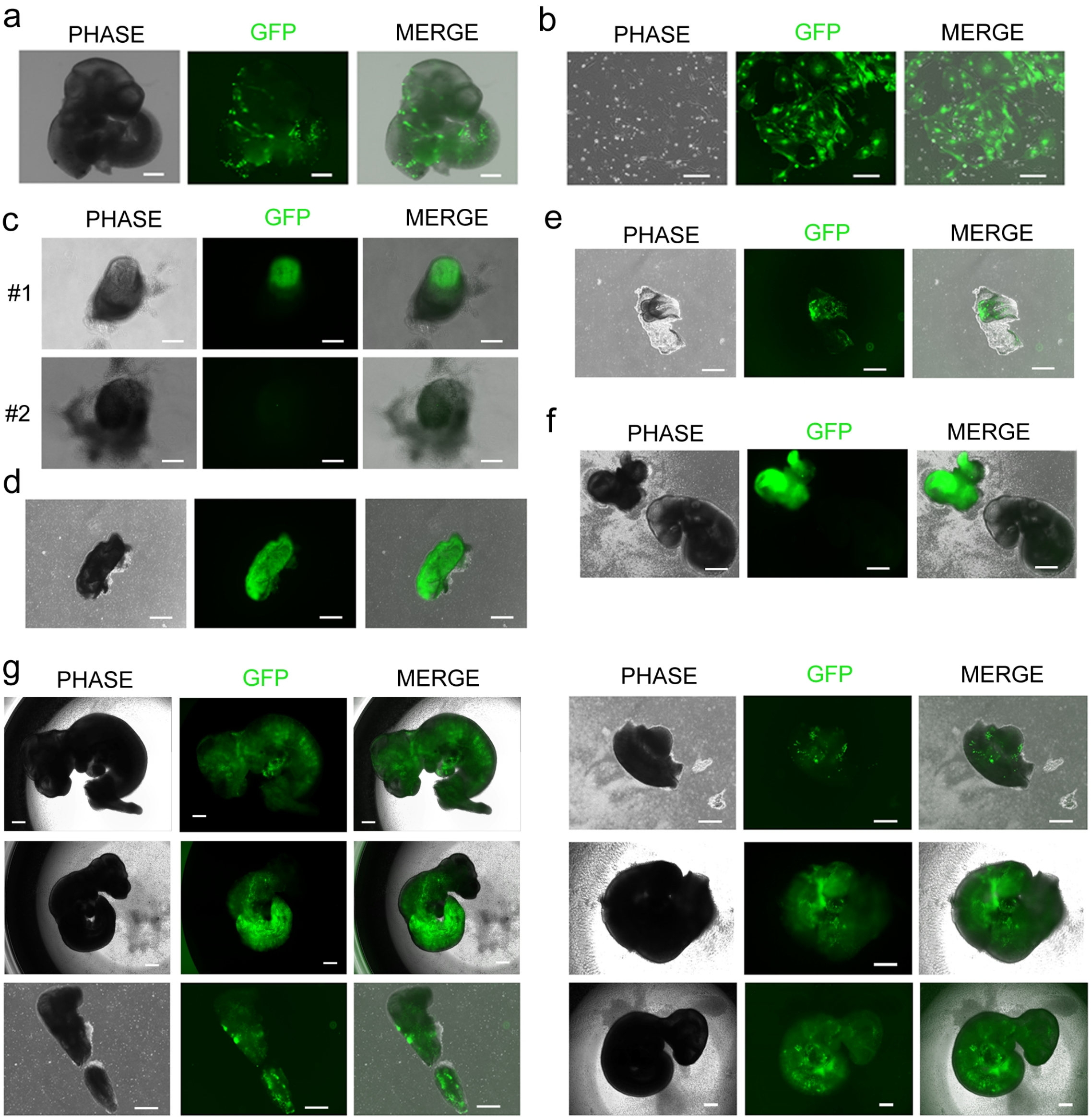

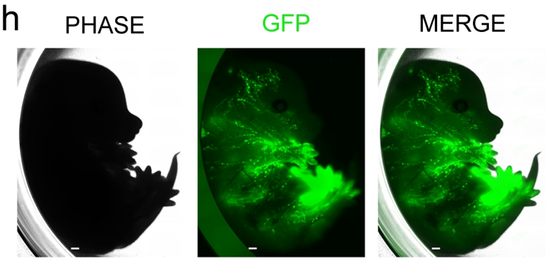

i

Line	Embryos recovered	Chimeric embryos	%Chimeric embryos
B6D2F1	21	7	33%
129P2/Ola	17	7	41%

Fig. S5 Chimeric contribution of GFP-labeled 6iL-mESCs.

a Representative fluorescence images of E9.5 chimaeras from blastocyst injected with GFP labeled 6iL-mESCs. Scale bars, 500 μm.

b Representative fluorescence images of GFP^+ cells cultured from dissociated E9.5 chimeric embryos presented in (a). Scale bars, 100 μm.

c Representative fluorescence images of E6.5 chimeric embryos. #1, chimeric embryo generated by blastocyst injection of GFP-labeled 6iL-mESCs; #2, control embryo generated from blastocysts without mESC injection. Scale bar, 200 μm

d Fluorescence images of E7 chimeric embryos generated by blastocyst injection of GFP-labeled 6iL-mESCs. Scale bars, 500 μm.

e Fluorescence images of E7.5 chimeric embryo generated by blastocyst injection of GFP-labeled 6iL-mESCs. Scale bars, 500 μm.

f Fluorescence images of E8.5 chimeric embryos generated by blastocyst injection of GFP-labeled 6iL-mESCs and an E9.5 WT embryo. Scale bars, 500 μm.

g Fluorescence images of E9.5 chimeric embryos from blastocyst injected with GFP labeled 6iL-mESCs. Scale bars, 500 μm.

h Fluorescence images of an E13.5 chimeric embryo generated by blastocyst injection of GFP labeled 6iL-mESCs. Scale bars, 500 μm.

i Summary of the number of chimeric embryos among recovered embryos.
