# Supplementary material for: A universal 6iL/E4 culture system for deriving and maintaining embryonic stem cells across mammalian species
Source: Cell Res. 2026 Jul 13;36(8):611–28. doi: 10.1038/s41422-026-01276-y (PMC13424318; doi:10.1038/s41422-026-01276-y)
Supplement: Supplementary file 6 — Supplementary information, Fig. S6 [file 41422_2026_1276_MOESM6_ESM.pdf]

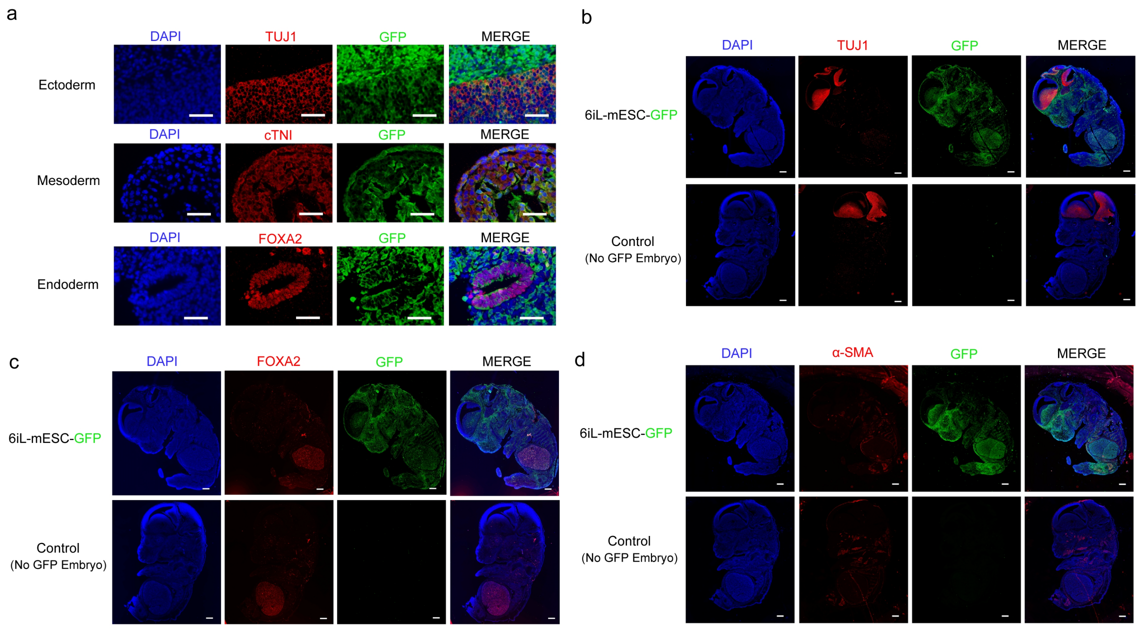

Fig. S6 Contribution of GFP-labeled 6iL-mESCs to embryonic germ layers in chimeric embryos.

a Representative images of sections from E9.5 embryos derived from blastocysts injected with GFP-labeled 6iL-mESCs, co-stained for GFP and lineage markers of the endoderm (FOXA2), mesoderm (cTNI), and ectoderm (TUJ1), to assess the contribution of 6iL-mESC-derived cells to distinct embryonic germ layers. Scale bars, 50 μm.

b Representative images of sections from E13.5 embryos derived from blastocysts injected with GFP-labeled 6iL-mESCs and stage-matched control embryos, co-stained for GFP and the ectoderm marker TUJ1. Scale bars, 500 μm.

c Representative section from E13.5 embryos derived from blastocysts injected with GFP-labeled 6iL-mESCs and stained for GFP and the endoderm marker FOXA2. Scale bars, 500 μm.

d Representative section from E13.5 embryos derived from blastocysts injected with GFP-labeled 6iL-mESCs and stained for GFP and the mesoderm marker α-SMA. Scale bars, 500 μm.
