# Supplementary material for: A universal 6iL/E4 culture system for deriving and maintaining embryonic stem cells across mammalian species
Source: Cell Res. 2026 Jul 13;36(8):611–28. doi: 10.1038/s41422-026-01276-y (PMC13424318; doi:10.1038/s41422-026-01276-y)
Supplement: Supplementary file 7 — Supplementary information, Fig. S7 [file 41422_2026_1276_MOESM7_ESM.pdf]

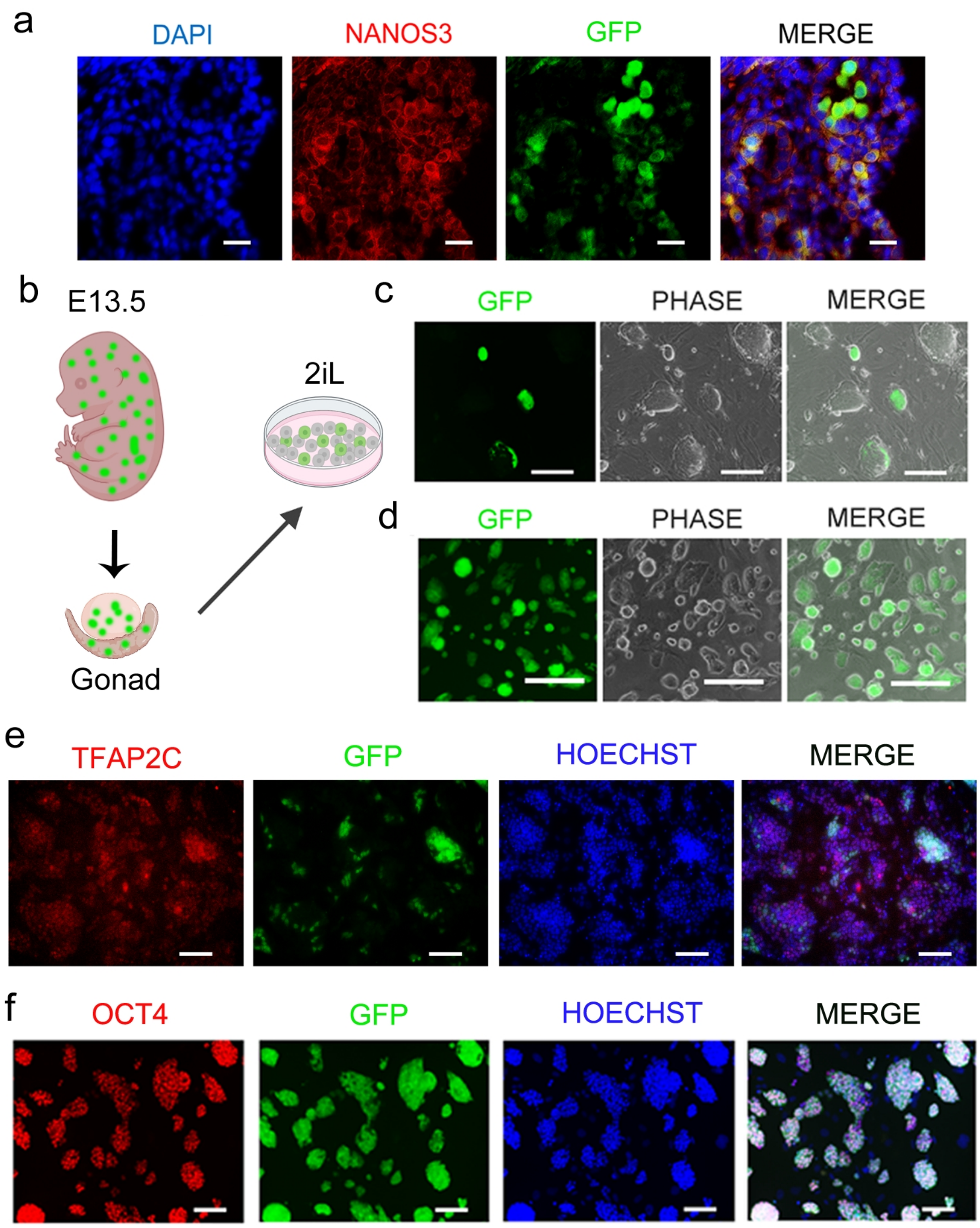
a
DAPI
NANOS3
GFP
MERGE
b
E13.5
Gonad
2iL
c
GFP
PHASE
MERGE
d
GFP
PHASE
MERGE
e
TFAP2C
GFP
HOECHST
MERGE
f
OCT4
GFP
HOECHST
MERGE

Fig. S7 Contribution of GFP-labeled 6iL-mESC-derived cells in chimeric gonads.

a Representative fluorescence images of gonadal sections from E13.5 chimeric embryos generated by blastocyst injection of GFP-labeled 6iL-mESCs. Sections were stained for NANOS3 (red) to identify PGCs, GFP (green) to trace 6iL-mESC-derived cells, and DAPI (blue) to label nuclei. Merged images show co-localization of GFP and NANOS3 signals within the gonad. Scale bars, 20 μm.

b Schematic illustration of isolation of gonads from E13.5 chimeric embryos followed by culture under 2iL conditions to derive PGC cells (created with BioRender.com).

c Representative phase-contrast and fluorescence images of GFP^+ colonies derived from the gonads of E13.5 chimeric embryos (chimaeras from blastocyst injected with GFP labeled 6iL-mESC) cultured in 2iL. Quantification of EGC cultures showed that 27.8% (17/61) of the colonies were GFP^+. Scale bars, 200 μm.

d Representative phase-contrast and fluorescence images of passage 3 EGCs derived from GFP^+ colony shown in (**c**), cultured under 2i/LIF conditions. Scale bars, 200 μm.

e IF results confirming the presence of cells showing co-localization of GFP and TFAP2C in EGC cells derived from isolated and cultured chimeric gonads. Scale bars, 200 μm.

f IF results confirming OCT4(red) expression in the GFP^+ EGCs in (**d**). Scale bars, 50 μm.
