# Supplementary material for: A universal 6iL/E4 culture system for deriving and maintaining embryonic stem cells across mammalian species
Source: Cell Res. 2026 Jul 13;36(8):611–28. doi: 10.1038/s41422-026-01276-y (PMC13424318; doi:10.1038/s41422-026-01276-y)
Supplement: Supplementary file 8 — Supplementary information, Fig. S8 [file 41422_2026_1276_MOESM8_ESM.pdf]

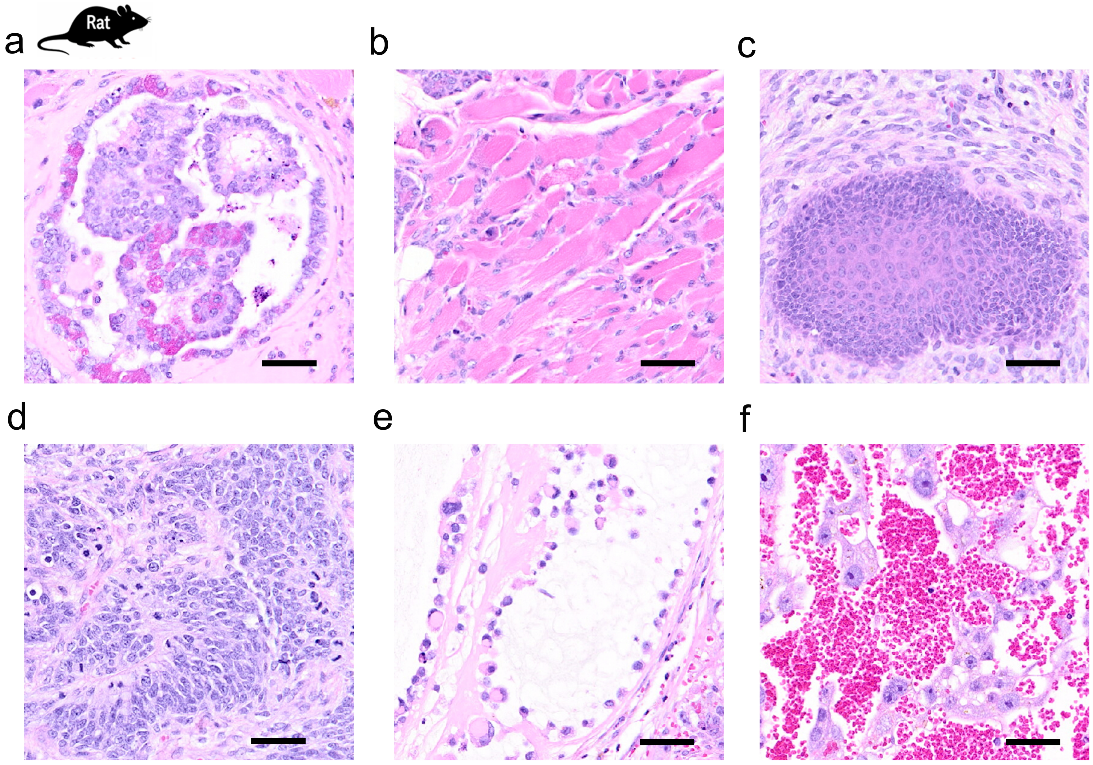

Fig. S8 Representative H&E staining results of rat teratomas derived from 6iL-rESCs.

a Intestine-like glandular epithelium with Paneth cell-like pink granules (endoderm). Scale bars, 50μm.

b Skeletal muscle (mesoderm). Scale bars, 50μm.

c Squamous epithelium (ectoderm). Scale bars, 50μm.

d Primitive neuroectoderm (ectoderm). Scale bars, 50μm.

e Yolk sac-like epithelium (extraembryonic endodermal tissues). Scale bars, 50μm.

f Giant trophoblast-like cells (extraembryonic endodermal tissues). Scale bars, 50μm.
