# Supplementary material for: A universal 6iL/E4 culture system for deriving and maintaining embryonic stem cells across mammalian species
Source: Cell Res. 2026 Jul 13;36(8):611–28. doi: 10.1038/s41422-026-01276-y (PMC13424318; doi:10.1038/s41422-026-01276-y)
Supplement: Supplementary file 9 — Supplementary information, Fig. S9 [file 41422_2026_1276_MOESM9_ESM.pdf]

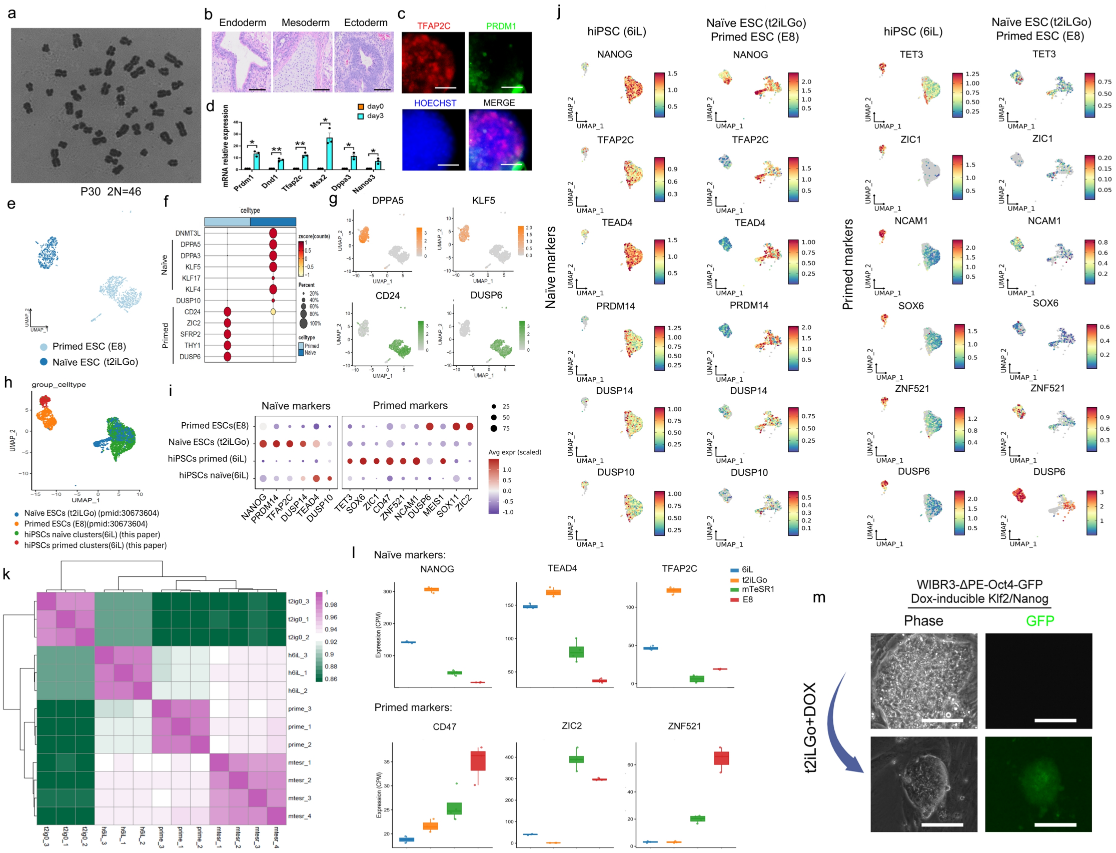

a
P30 2N=46
b
Endoderm
Mesoderm
Ectoderm
c
TFAP2C
PRDM1
HOECHST
MERGE
d
mRNA relative expression
day0
day3
Prdm1
Dnd1
Tfap2c
Msx2
Dppa3
Nanos3
e
Primed ESC (E8)
Naïve ESC (t2iLGo)
f
celltype
Naive
Primed
DNMT3L
DPPA5
DPPA3
KLF5
KLF17
KLF4
DUSP10
CD24
ZIC2
SFRP2
THY1
DUSP6
g
DPPA5
KLF5
CD24
DUSP6
h
group_celltype
Naïve ESCs (t2iLGo) (pmid:30673604)
Primed ESCs (E8)(pmid:30673604)
hiPSCs naive clusters(6iL) (this paper)
hiPSCs primed clusters(6iL) (this paper)
i
Naïve markers
Primed markers
Primed ESCs(E8)
Naïve ESCs (t2iLGo)
hiPSCs primed (6iL)
hiPSCs naïve(6iL)
Avg expr (scaled)
j
hiPSC (6iL)
Naïve ESC (t2iLGo)
Primed ESC (E8)
Naïve markers
Primed markers
NANOG
TFAP2C
TEAD4
PRDM14
DUSP14
DUSP10
TET3
ZIC1
NCAM1
SOX6
ZNF521
DUSP6
k
l
Naïve markers:
NANOG
TEAD4
TFAP2C
6iL
t2iLGo
mTeSR1
E8
Primed markers:
CD47
ZIC2
ZNF521
Expression (CPM)
m
WIBR3-ΔPE-Oct4-GFP
Dox-inducible Klf2/Nanog
Phase
GFP
t2iLGo+DOX

Fig. S9 Characterization of pluripotency features and transcriptional identity of 6iL-cultured human PSCs.

a Representative karyotype of 6iL-hESCs at passage 30 (2N = 46).

b Representative H&E staining of teratomas derived from 6iL-hESCs, showing differentiated tissues representative of the three germ layers: endoderm, mesoderm, and ectoderm. Scale bars, 100 μm.

c Immunofluorescence staining of TFAP2C and PRDM1 in EBs formed from 6iL-hESCs. Scale bars, 50 μm.

d qRT-PCR analysis of PGC marker gene expression on days 0 and 3 of PGC-LCs differentiated from 6iL-hiPSCs. Data are presented as mean ± SEM. *p < 0.05, **p < 0.01.

e UMAP projection of single-cell RNA-seq data showing clustering of primed hESCs cultured in E8 conditions (light blue, 814 cells) and naïve ESCs cultured in t2iLGo conditions (dark blue, 436 cells), illustrating transcriptional separation between primed and naïve pluripotent states.

f Dot plot showing expression of representative naïve and primed pluripotency markers across primed and naïve hESCs shown in panel (**e**). Dot size indicates the percentage of cells expressing each gene, and color intensity represents scaled expression levels (z-score of counts). Naïve-associated genes (*DNMT3L*, *DPPA5*, *DPPA3*, *KLF5*, *KLF17*, *KLF4*, and *DUSP10*) are enriched in naïve hESCs (t2iLGo), whereas primed-associated genes (*CD24*, *ZIC2*, *SFRP2*, *THY1*, and *DUSP6*) are predominantly expressed in primed hESCs (E8).

g Feature plots showing expression of representative naïve-associated genes (*DPPA5* and *KLF5*) and primed-associated genes (*CD24* and *DUSP6*) projected onto the UMAP embedding. Naïve markers are predominantly expressed in the naïve hESC(t2iLGo) cluster, whereas primed markers are enriched in the primed hESC (E8) population.

h Integrated UMAP visualization showing transcriptional relationships among naïve hESCs (t2iLGo), primed hESCs (E8), hiPSC naïve clusters (6iL), and hiPSC primed clusters (6iL) from this study.

i Dot plot summarizing expression of representative naïve and primed pluripotency markers across primed hESCs (E8), naïve hESCs (t2iLGo), and hiPSC populations cultured under 6iL conditions. Dot size indicates the fraction of cells expressing each gene, and color intensity represents scaled average expression levels.

j UMAP feature plots showing expression patterns of representative naïve-associated genes (*NANOG*, *TFAP2C*, *TEAD4*, *PRDM14*, *DUSP14*, and *DUSP10*) and primed-associated genes (*TET3*, *ZIC1*, *NCAM1*, *SOX6*, *ZNF521*, and *DUSP6*) in hiPSCs cultured under 6iL conditions and reference naïve (t2iLGo) and primed (E8) ESC populations. Gene expression levels are indicated by color intensity, illustrating differential enrichment of naïve and primed transcriptional programs across pluripotent stem cell states.

k Heatmap showing pairwise sample correlation based on bulk RNA-seq gene expression profiles across pluripotent stem cell states. Samples cultured under t2iLGo, 6iL (Passage 25), primed (E8), and mTeSR conditions cluster according to transcriptional similarity. Color scale represents Pearson correlation coefficients calculated from normalized gene expression values.

l Quantification of representative naïve and primed pluripotency marker expression based on bulk RNA-seq data shown in panel (**k**). Counts per million (CPM) of naïve markers (*NANOG*, *TEAD4*, and *TFAP2C*) and primed markers (*CD47*, *ZIC2*, and *ZNF521*) are compared across

PSCs cultured under 6iL, t2iLGo, mTeSR1, and E8 conditions, illustrating differential activation of naïve and primed transcriptional programs.

m WIBR3-ΔPE-Oct4-GFP cells carrying Dox-inducible Klf2/Nanog were cultured under t2iLGo+DOX conditions. Phase contrast (left) and GFP fluorescence (right) images are shown. Scale bars=100μm.
