# Supplementary material for: A universal 6iL/E4 culture system for deriving and maintaining embryonic stem cells across mammalian species
Source: Cell Res. 2026 Jul 13;36(8):611–28. doi: 10.1038/s41422-026-01276-y (PMC13424318; doi:10.1038/s41422-026-01276-y)
Supplement: Supplementary file 10 — Supplementary information, Fig. S10 [file 41422_2026_1276_MOESM10_ESM.pdf]

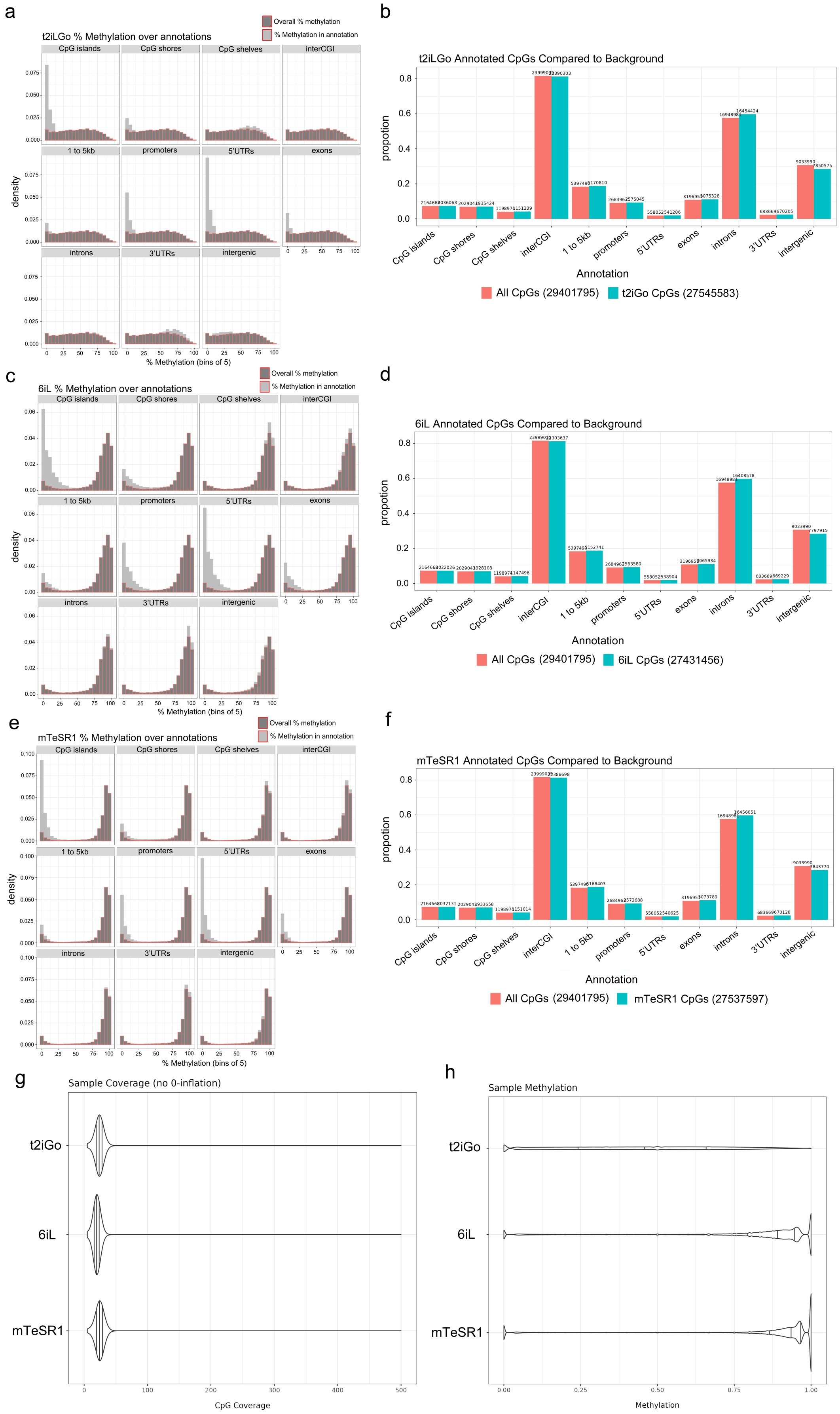

a
t2iLGo % Methylation over annotations
Overall % methylation
% Methylation in annotation
CpG islands
CpG shores
CpG shelves
interCGI
1 to 5kb
promoters
5'UTRs
exons
introns
3'UTRs
intergenic
density
% Methylation (bins of 5)
b
t2iLGo Annotated CpGs Compared to Background
propotion
Annotation
All CpGs (29401795)
t2iGo CpGs (27545583)
c
6iL % Methylation over annotations
d
6iL Annotated CpGs Compared to Background
All CpGs (29401795)
6iL CpGs (27431456)
e
mTeSR1 % Methylation over annotations
f
mTeSR1 Annotated CpGs Compared to Background
All CpGs (29401795)
mTeSR1 CpGs (27537597)
g
Sample Coverage (no 0-inflation)
t2iGo
6iL
mTeSR1
CpG Coverage
h
Sample Methylation
Methylation

Fig. S10 Genome-wide DNA methylation profiling of PSCs cultured under different conditions revealed by WGBS.

a Distribution of CpG methylation levels across genomic annotations in DOX-induced Klf2/Nanog-reset naïve hESCs cultured under t2iL/Go conditions. Histograms show methylation density across CpG islands, CpG shores, CpG shelves, inter-CGI regions, promoters, gene bodies, untranslated regions (UTRs), introns, and intergenic regions. Grey bars represent overall CpG methylation.

b Proportional distribution of annotated CpGs in t2iLGo-hESCs compared with genomic background CpGs. Bars represent the fraction of CpGs located within each genomic annotation category.

c Distribution of CpG methylation levels across genomic annotations in hiPSCs cultured under 6iL conditions, displayed as in panel (**a**).

d Proportional distribution of annotated CpGs in 6iL-hiPSCs compared with genomic background CpGs.

e Distribution of CpG methylation levels across genomic annotations in hiPSCs cultured under mTeSR1 conditions, displayed as in panel (**a**).

f Proportional distribution of annotated CpGs in mTeSR1-hiPSCs compared with genomic background CpGs.

g Violin plots showing CpG coverage distribution across samples following WGBS analysis (excluding zero-inflated regions).

h Genome-wide methylation level distributions across samples cultured under t2iLGo, 6iL, and mTeSR1 conditions.
