# Supplementary material for: A universal 6iL/E4 culture system for deriving and maintaining embryonic stem cells across mammalian species
Source: Cell Res. 2026 Jul 13;36(8):611–28. doi: 10.1038/s41422-026-01276-y (PMC13424318; doi:10.1038/s41422-026-01276-y)
Supplement: Supplementary file 11 — Supplementary information, Fig. S11 [file 41422_2026_1276_MOESM11_ESM.pdf]

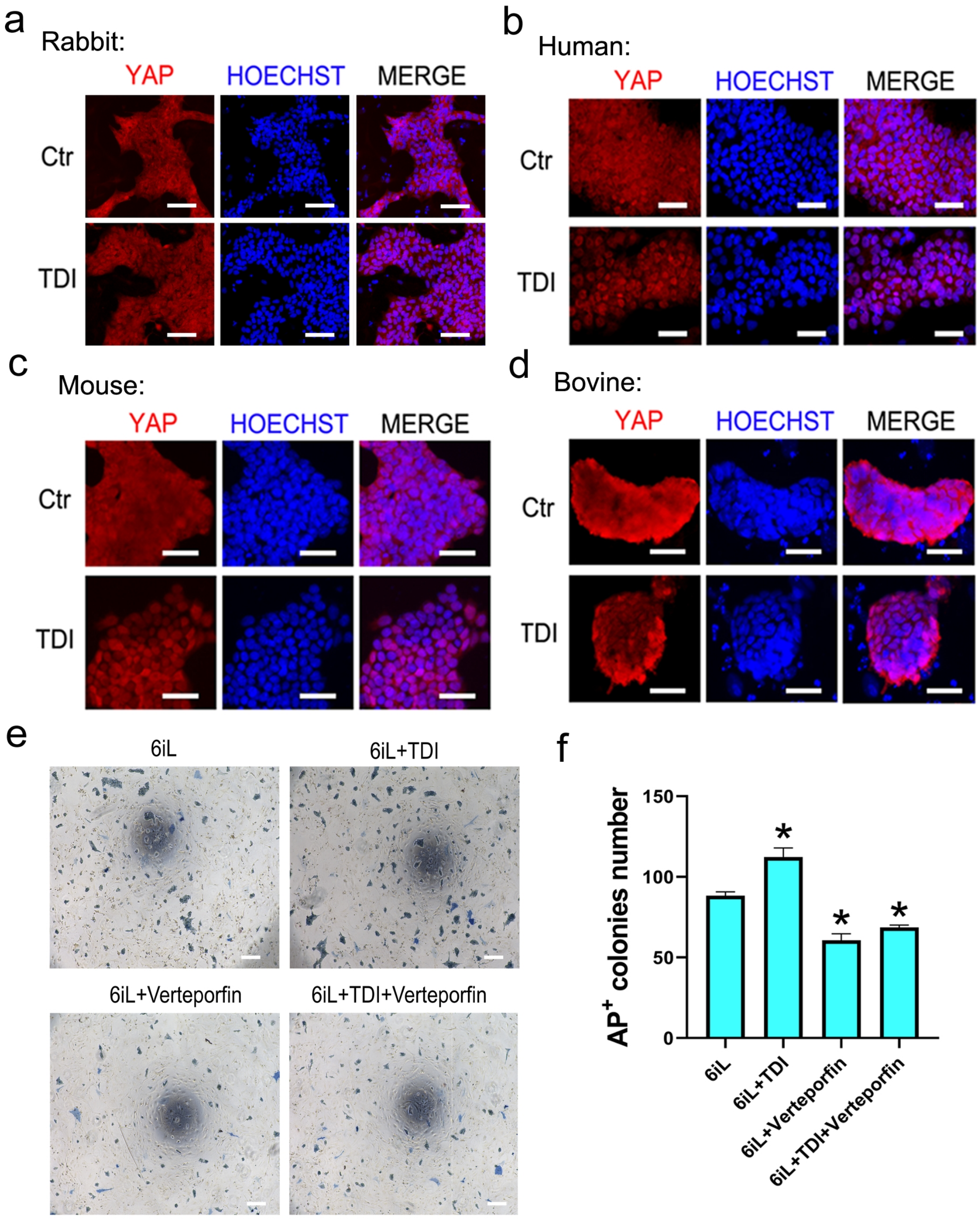
a
Rabbit:
YAP
HOECHST
MERGE
Ctr
TDI
b
Human:
YAP
HOECHST
MERGE
Ctr
TDI
c
Mouse:
YAP
HOECHST
MERGE
Ctr
TDI
d
Bovine:
YAP
HOECHST
MERGE
Ctr
TDI
e
6iL
6iL+TDI
6iL+Verteporfin
6iL+TDI+Verteporfin
f
AP+ colonies number
150
100
50
0
*
6iL
6iL+TDI
6iL+Verteporfin
6iL+TDI+Verteporfin

Fig. S11 TDI modulates YAP localization and ESC colony formation.

a–d Immunofluorescence staining showing YAP localization (red) in ESCs from rabbit (**a**), human (**b**), mouse (**c**), and bovine (**d**) cultured under control (Ctr) or TDI conditions. Nuclei were counterstained with Hoechst (blue). TDI treatment alters YAP localization compared with control conditions across multiple species. Scale bars, 50 μm.

e Representative images of AP staining showing colony morphology of rabESCs cultured under 6iL, 6iL + TDI, 6iL + Verteporfin, or 6iL + TDI + Verteporfin conditions. Scale bars, 300μm.

f Quantification of AP-positive colony numbers under the indicated culture conditions shown in (e). Data are presented as mean ± SEM. $P < 0.05$.
