# Supplementary material for: A universal 6iL/E4 culture system for deriving and maintaining embryonic stem cells across mammalian species
Source: Cell Res. 2026 Jul 13;36(8):611–28. doi: 10.1038/s41422-026-01276-y (PMC13424318; doi:10.1038/s41422-026-01276-y)
Supplement: Supplementary file 12 — Supplementary information, Fig. S12 [file 41422_2026_1276_MOESM12_ESM.pdf]

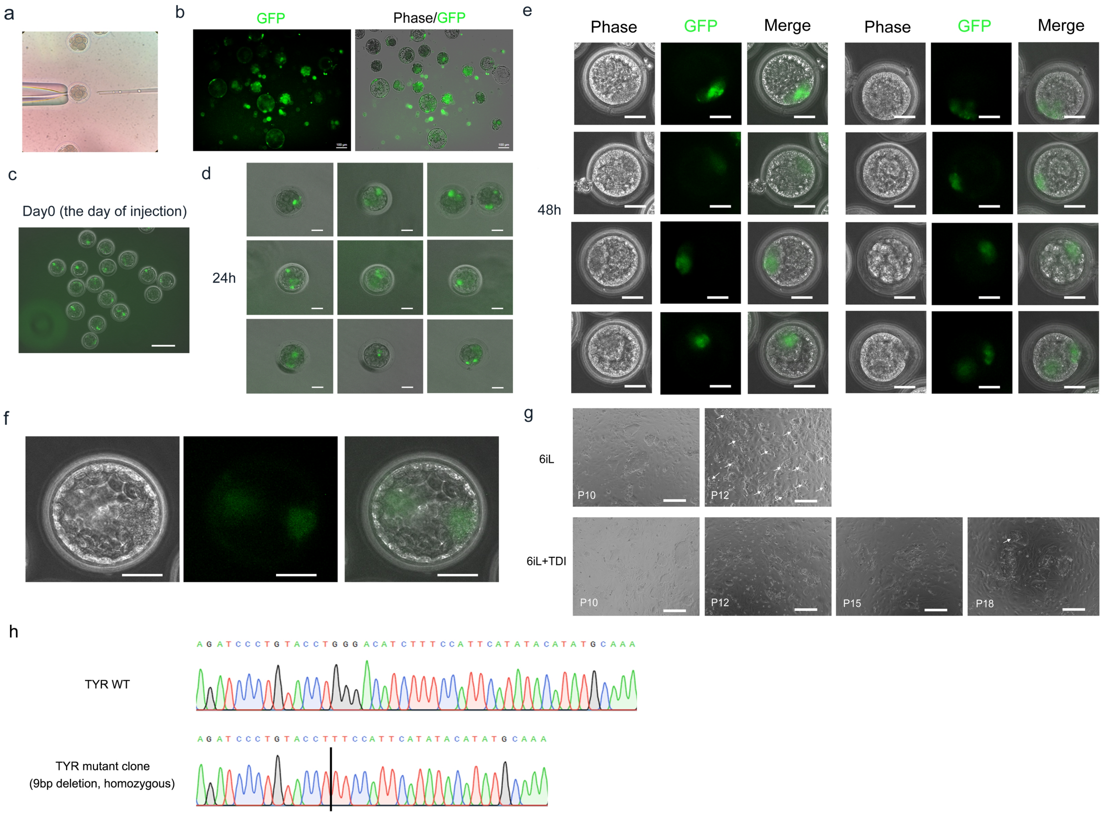

Fig. S12 Functional integration and expansion capacity of GFP-labeled 6iL/TDI-rabESCs in rabbit embryos.

a Microinjection of GFP-labeled 6iL rabESCs into 8-cell stage rabbit embryos.

b Blastocysts were generated in vitro from 8-cell stage rabbit embryos injected with GFP-labeled 6iL/TDI-rabESCs. Scale bars, 100 μm.

c Representative images of injected 8-cell stage embryos on day 0 (the day of injection), showing GFP-labeled 6iL/TDI rabESCs within embryos immediately after microinjection. Scale bars, 200 μm.

d Time-course images showing survival and localization of GFP-labeled 6iL/TDI rabESCs within embryos 24h after injection. Scale bars, 50 μm.

e Representative embryos at 48 h post-injection showing persistence and distribution of GFP-labeled 6iL/TDI rabESCs during *in vitro* development. Scale bars, 50 μm.

f A representative blastocyst-stage embryo showing GFP^{+} cell contribution following injection of GFP-labeled 6iL/TDI rabESCs at the 8-cell stage and subsequent *in vitro* development. Scale bars, 50 μm.

g Representative morphology of rabESCs derived from morula-stage embryos in 6iL or 6iL/TDI at different passages. Arrows indicate differentiated cells. Scale bars, 200 μm.

h Sanger sequencing analysis showing genome-editing outcomes at the *Tyr* locus in 6iL-rabESC monoclonal lines compared with wild-type (WT) cells, including representative deletion events.
