# Supplementary material for: A universal 6iL/E4 culture system for deriving and maintaining embryonic stem cells across mammalian species
Source: Cell Res. 2026 Jul 13;36(8):611–28. doi: 10.1038/s41422-026-01276-y (PMC13424318; doi:10.1038/s41422-026-01276-y)
Supplement: Supplementary file 13 — Supplementary information, Fig. S13 [file 41422_2026_1276_MOESM13_ESM.pdf]

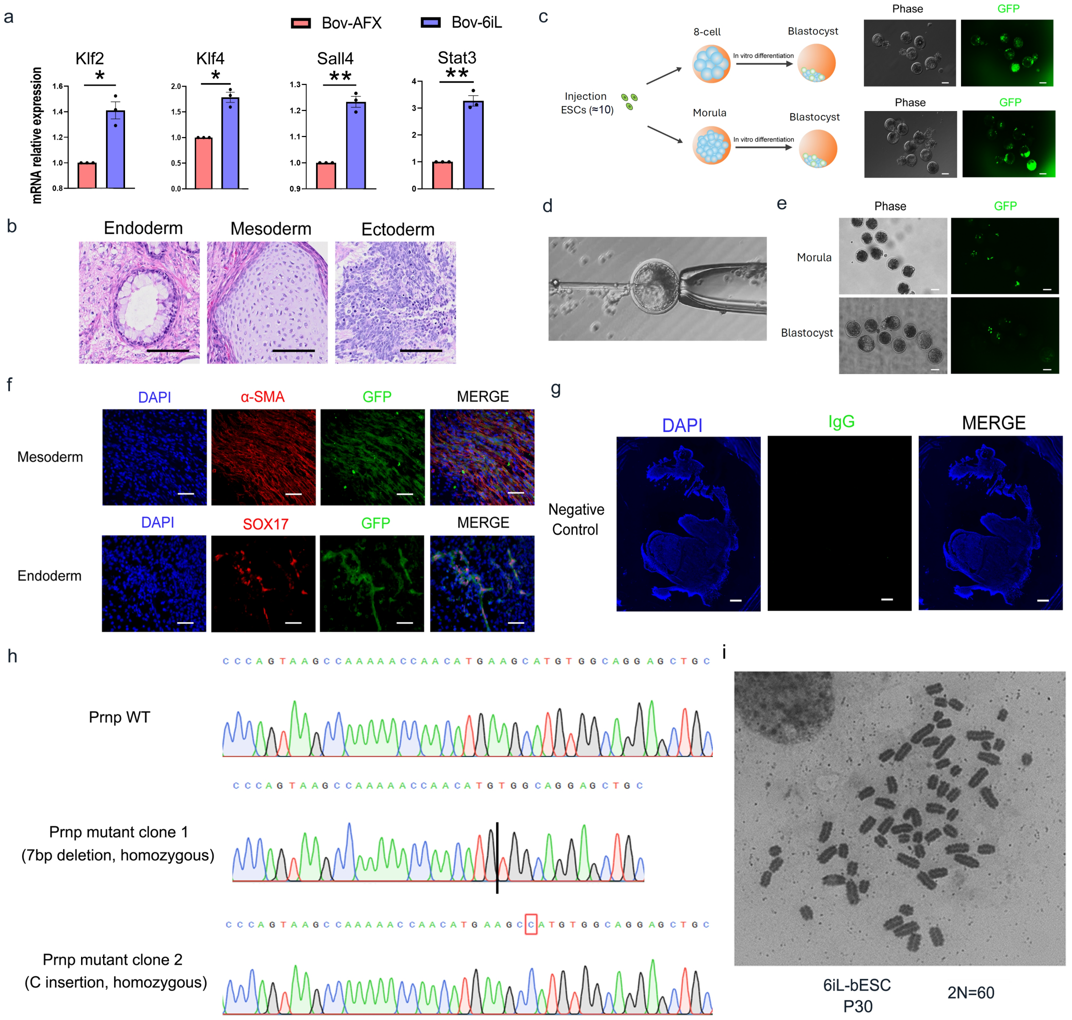
a
Bov-AFX
Bov-6iL
Klf2
Klf4
Sall4
Stat3
mRNA relative expression
b
Endoderm
Mesoderm
Ectoderm
c
Injection ESCs (≈10)
8-cell
Morula
In vitro differentiation
Blastocyst
Phase
GFP
d
e
Morula
Blastocyst
f
DAPI
α-SMA
SOX17
GFP
MERGE
Mesoderm
Endoderm
g
IgG
Negative Control
h
Prnp WT
Prnp mutant clone 1
(7bp deletion, homozygous)
Prnp mutant clone 2
(C insertion, homozygous)
CCCAGTAAGCCAAAAACCAACATGAAGCATGTGGCAGGAGCTGC
CCCAGTAAGCCAAAAACCAACATGTGGCAGGAGCTGC
CCCAGTAAGCCAAAAACCAACATGAAGCCATGTGGCAGGAGCTGC
i
6iL-bESC
P30
2N=60

Fig. S13 Characterization and developmental contribution of 6iL-bESCs.

a qRT–PCR analysis of pluripotency-associated genes (*Klf2, Klf4, Sall4,* and *Stat3*) in bESCs cultured in either AFX or 6iL. Gene expression levels are shown as relative mRNA expression. Data are presented as mean ± SEM. *, $P < 0.05$; **, $P < 0.01$.

b Representative H&E staining of teratomas derived from 6iL-hESCs, showing differentiated tissues representative of the three germ layers: endoderm, mesoderm, and ectoderm. Scale bars, 100 μm.

c Schematic representation of the injection of GFP-labeled bESCs (8-10 cells) into 8-cell and morula-stage bovine embryos, followed by *in vitro* culture to the blastocyst stage. Representative phase-contrast and GFP fluorescence images show the presence of GFP^+ cells in blastocysts. Scale bars, 100 μm.

d Microinjection of GFP-labeled ESCs into a bovine blastocyst.

e Representative phase-contrast and GFP fluorescence images showing GFP-positive ESCs in morula- and blastocyst-stage embryos following injection and *in vitro* culture. Scale bars, 100 μm.

f Immunofluorescence staining of sections from E40 chimeric bovine embryos generated using GFP-labeled 6iL-bESCs. Mesoderm (α-SMA, red) and endoderm (SOX17, red) markers are shown together with GFP (green) to identify 6iL-bESC-derived cells and DAPI nuclear staining (blue). Merged images demonstrate contribution of GFP-labeled 6iL-bESC-derived cells to multiple embryonic lineages. Scale bars, 50 μm.

g Negative control immunostaining performed using an IgG antibody derived from the same host species as the anti-GFP antibody together with the same fluorescent secondary antibody used for GFP staining, showing the absence of nonspecific signal. Nuclei were counterstained with DAPI (blue). Scale bars, 1000 μm.

h Sanger sequencing analysis showing genome-editing outcomes at the bovine *Prnp* locus in 6iL-bESC monoclonal lines compared with WT cells, including representative insertion and deletion events in *Prnp*.

i Representative karyotype of 6iL-bESCs at passage 30.
