# Supplementary material for: A universal 6iL/E4 culture system for deriving and maintaining embryonic stem cells across mammalian species
Source: Cell Res. 2026 Jul 13;36(8):611–28. doi: 10.1038/s41422-026-01276-y (PMC13424318; doi:10.1038/s41422-026-01276-y)
Supplement: Supplementary file 14 — Supplementary information, Table S1 [file 41422_2026_1276_MOESM14_ESM.pdf]

Supplementary information, Table S1.

Proportion of rabbit and bovine embryos that formed proliferative inner cell mass clones under different culture conditions.

	BRD0705/IWR1	CHIR	BRD0705/IWR1/LIF	CHIR/LIF
Bovine	28.6% (4/14)	0 (0/12)	42.9% (5/14)	16.7% (2/12)
Rabbit	20% (3/15)	0 (0/15)	33.3% (4/15)	13.3% (2/15)
	BRD0705/IWR1/LIF/828+PD0325901	BRD0705/IWR1/LIF/828 +SU5402/PD184	BRD0705/IWR1/LIF/828+SU5402	
Bovine	18.2% (2/11)	46.2% (6/13)	45.5% (5/11)	
Rabbit	7.7% (1/13)	50% (6/12)	50% (6/12)	
