# Supplementary material for: A universal 6iL/E4 culture system for deriving and maintaining embryonic stem cells across mammalian species
Source: Cell Res. 2026 Jul 13;36(8):611–28. doi: 10.1038/s41422-026-01276-y (PMC13424318; doi:10.1038/s41422-026-01276-y)
Supplement: Supplementary file 15 — Supplementary information, Table S2 [file 41422_2026_1276_MOESM15_ESM.pdf]

Supplementary information, Table S2.

Number of embryos forming ESC colonies after one passage following derivation from blastocyst-stage (bovine) and morula-stage (rabbit) embryos relative to the total number of embryos.

Passage1	BRD0705/IWR1/LIF/828+SU5402	BRD0705/IWR1/LIF/828 +Axitinib	BRD0705/IWR1/LIF/828 + Futibatinib	BRD0705/IWR1/LIF/828 +CP673451
Bovine	45.5% (5/11)	0 (0/10)	11.1% (1/9)	60% (6/10)
Rabbit	50% (6/12)	8.3% (1/12)	0 (0/9)	58.3% (7/12)
