# Supplementary material for: A universal 6iL/E4 culture system for deriving and maintaining embryonic stem cells across mammalian species
Source: Cell Res. 2026 Jul 13;36(8):611–28. doi: 10.1038/s41422-026-01276-y (PMC13424318; doi:10.1038/s41422-026-01276-y)
Supplement: Supplementary file 16 — Supplementary information, Table S3 [file 41422_2026_1276_MOESM16_ESM.pdf]

Supplementary information, Table S3.

Number of embryos forming ESC colonies at passages 1 and 3 following derivation from blastocyst-stage (bovine) and morula-stage (rabbit) embryos, relative to the total number of embryos.

Passage 1	BRD0705/IWR1/CP673451/LIF/828+PD184	BRD0705/IWR1/CP673451/LIF/828+ GDC0879	BRD0705/IWR1/CP673451/LIF/828+ Vx11e	BRD0705/IWR1/ CP673451/LIF/828+JNK-IN-8
Bovine	10% (1/10)	88% (16/18)	0 (0/8)	25% (2/8)
Rabbit	0 (0/12)	75% (12/16)	0 (0/7)	44.4% (4/9)
Passage 3	BRD0705/IWR1/CP673451/LIF/828+PD184	BRD0705/IWR1/CP673451/LIF/828+ GDC0879	BRD0705/IWR1/CP673451/LIF/828+ Vx11e	BRD0705/IWR1/ CP673451/LIF/828+JNK-IN-8
Bovine	0 (0/10)	77% (14/18)	0 (0/8)	12% (1/8)
Rabbit	0 (0/12)	62% (10/16)	0 (0/7)	11% (1/9)
