# Supplementary material for: A universal 6iL/E4 culture system for deriving and maintaining embryonic stem cells across mammalian species
Source: Cell Res. 2026 Jul 13;36(8):611–28. doi: 10.1038/s41422-026-01276-y (PMC13424318; doi:10.1038/s41422-026-01276-y)
Supplement: Supplementary file 17 — Supplementary information, Table S4 [file 41422_2026_1276_MOESM17_ESM.pdf]

Supplementary information, Table S4.

List of qPCR primers.

**Rat**	**FW**	**RV**
Prdm1	AGTGTAACTCCGGTTCCGTG	GAACAGAAGTACCCCCGTCG
Tfap2c	CGCATCCCTCACCTCTCATC	TAATGATCGGCGGATTGCGA
Utf1	CCTGCAGTGGTGGAGCAAGA	CGGGGAGGATTCGAGGGTAT
Dnd1	ACCTTTAGTGGCCTGAACCG	GCTCGCACTTCTCGGTACTT
Nanos3	GTCTACTGCTACACCACCCG	TTGGAACCCGCATAGACACC
Msx2	CTCTCGTCAAGCCCTTCGAG	CTAGAAGCTGGGACGTGGTG
Gapdh	TGGTCATCAACGGGAAACCC	GAAGGGGCGGAGATGATGAC
**Bovine**	**FW**	**RV**
Nanog	GCAGAAAAACAACTGGCCGA	ACCCCTGGTGGTAGGAATAGA
Pou5f1	CTCGGACCTGGATGAGCTTC	TCCACACAAGTCATAGGGCG
Sox2	GCGCCGAGTGGAAACTTTTG	CATGAGCGTCTTGGTTTTCCG
Rex1	TCCCGGAGGAATACCCAACA	CTTGTGCATCCGTTGTGAGG
Otx2	GGAGAGGACGACATTCACCC	TACCCTGGACTCAGGCAAGT
Esrrb	AAGCACATCCCAGGCTTCTC	ACACCAGCTTGTCGTCGTAG
Prdm1	GGAGGACGCGGATATGACTC	CCAGGGGTGGTCATTCACAA
Kit	CTCGTCCAGACAGGCTCTTC	CAGCCTAATCTCGTCGCCAA
Dnd1	GGTGAACGGGCAGAGAAAGT	GACGAAACTCGTAGAGGCGG
Tfap2c	CGCCTGAGTGCTTAAATGCC	TGACATGAGCTGCTTTCCGT
Msx2	CACCCTTTACCACGTCCCAG	TCCTGCAGTCTTTTCGCCTT
Nanos3	TATGAACAAGGTTGTGTGGCG	CGAGGGACTTCCAGACACAAA
Sox17	ACAGTACCTGCACTTCGTGTG	ACACGTCGGGATAGTTGCAG
Gapdh	TCTGGCAAAGTGGACATCGT	TGATGACGAGCTTCCCGTTC
Klf2	CGTCCTTCTCCACTTTCGCTA	AACACGCTGTTGAGGTCGT
Klf4	TACCAAGAGCTCATGCCACC	CTTCGTGTAGGTTTTGCCGC
TFE3	GCCGACAGCAGATTGACGAT	CGTGGCTACGCCCTGATTAT
DUSP14	CGGCAGCTTTTTGGGAAGTC	CTCGTAAACGTCCGGCACTA
**Human**	**FW**	**RV**
Prdm1	AGCACTGTGAGGTTTCAGGG	CTGAACCGAAGTACCGCCAT
Dnd1	GTGAACGGGCAGAGGAAGTA	TGAAGGTCATCATCAGGCGG
Msx2	GCCTCGGTCAAGTCGGAAAA	GGCGCGCACTCACTTG
Dppa3	TGTTACTCGGCGGAGTTCG	ATGGCTGAAGTGGCTTGGTG
Nanos3	CACTTACTGGCCAGGGCTAC	GTCCTGTGTCTTCGCCTTGT
Tfcp2l1	AGCACTACAACCAGCACAACT	GGTTGAGGTAGGTCAGCGTC

ZFP42	TGGGAAAGCGTTCGTTGAGA	CACCCTTCAAAAGTGCACCG
Nanog	ATGGTGTGACGCAGGGATG	ACTGTTCCAGGCCTGATTGT
Pou5f1	AACCCACACTGCAGCAGATCA	GGCCAGAGGAAAGGACACTG
Gapdh	ATTCCATGGCACCGTCAAGG	TCGCCCCACTTGATTTTGGA
Dppa5	CCGGCACGTAGACATATCCC	GCCGAAAATGGCTTTCAGCA
Klf17	GAGATGGAACAGGAGGCTGG	TCTCTGCGCTGTGAGGAAAG
Gbx2	GGTTCCGGTCGGGGCT	GCTGTAGTCCACATCGCTCTC
**Mouse**	**FW**	**RV**
Gapdh	TTTGCAGTGGCAAAGTGGAGATT	CCCATTTGATGTTAGTGGGGTCTCG
Tfcp2l1	GGGGACTACTCGGAGCATCT	TGTTTCCGATCAGCTCCCTT
Oct4	GTGGACCTCAGGTTGGACTG	GGAGGTTCCCTCTGAGTTGC
Nanog	TGGAAGCCTTTCCATGTGGG	TATGGAGCGGAGCAGCATTC
Sox2	TTTGTCCGAGACCGAGAAGC	CTCCGGGAAGCGTGTACTTA
Otx2	CCCTGGGCTTCTTGTCCTG	CACCCTGGATTCTGGCAAGT
Foxa2	TGAAGATGGAAGGGCACGAG	CTCACGGAAGAGTAGCCCTC
Rex1	AAACGGCAAAGACAAGTGGC	TAGGGTCAGTCTGTCGAGGG
Gata4	AGCAGGACTCTTGGAACAGC	TACGCGGTGATTATGTCCCC
T	CTACATCCACCCAGACTCGC	CCCCCTCCATTGAGCTTGTT
Otx2	CCCTGGGCTTCTTGTCCTG	CACCCTGGATTCTGGCAAGT
Sox1	TCTCCAACTCTCAGGGCTACA	ACTTGACCAGAGATCCGAGG
Pax6	CAGATGCAAAAGTCCAGGTGC	CTTACTCCCTCCGATTGCCC
**Inducible vector**	**FW**	**RV**
Nanog	GCAAATGTCTTCTGCTGAGATGC	GTGGAAGAATCAGGGCTGTCCT
Klf2	GCGTGCTGGACTTCATCCTG	CGGGGTAATAGAACGCAGGC
Bovine Prnp targeting sgRNA	AAAACCAACATGAAGCATG	
Bovine Prnp-PCR-F	gccatgtggagtgacgtgg	
Bovine Prnp-PCR-R	cctgtagtacacttggttgggg	
Rabbit Tyr targeting sgRNA	uaugaauggaaagaugaucccc	
Rabbit Tyr-PCR-F	gctacaggagagagagcaaattggctcagc	
Rabbit Tyr-PCR-R	ccaagatctggctgagtctgagactttaactg	
