# Supplementary material for: A universal 6iL/E4 culture system for deriving and maintaining embryonic stem cells across mammalian species
Source: Cell Res. 2026 Jul 13;36(8):611–28. doi: 10.1038/s41422-026-01276-y (PMC13424318; doi:10.1038/s41422-026-01276-y)
Supplement: Supplementary file 18 — Supplementary information, Table S5 [file 41422_2026_1276_MOESM18_ESM.pdf]

**Supplementary information, Table S5.**

List of 6iL ESC derivation efficiency.

| Strain | Strain | Developmental stage | Embryo numbers | Derived cell lines | Efficiency % |
|---|---|---|---|---|---|
| mouse | B6D2F1 | Blastocyst | 16 | 12 | 75.00% |
| rabbit | | Blastocyst | 21 | 8 | 38.10% |
| rabbit | | morula | 25 | 17 | 68.00% |
| bovine | | Blastocyst | 45 | 39 | 86.67% |
