# Supplementary material for: A universal 6iL/E4 culture system for deriving and maintaining embryonic stem cells across mammalian species
Source: Cell Res. 2026 Jul 13;36(8):611–28. doi: 10.1038/s41422-026-01276-y (PMC13424318; doi:10.1038/s41422-026-01276-y)
Supplement: Supplementary file 23 — Supplementary information, Video legend [file 41422_2026_1276_MOESM23_ESM.pdf]

Supplementary information, Video S1. Beating cardiomyocytes differentiated from 6iL-rabESCs.
